# Supplementary material for: Identifying miRNA Signatures Associated with Pancreatic Islet Dysfunction in a FOXA2-Deficient iPSC Model
Source: Stem Cell Rev Rep. 2024 Jun 25;20(7):1915–31. doi: 10.1007/s12015-024-10752-0 (PMC11445299; doi:10.1007/s12015-024-10752-0)
Supplement: Supplementary file 1 — Supplementary Material 1 [file 12015_2024_10752_MOESM1_ESM.docx]

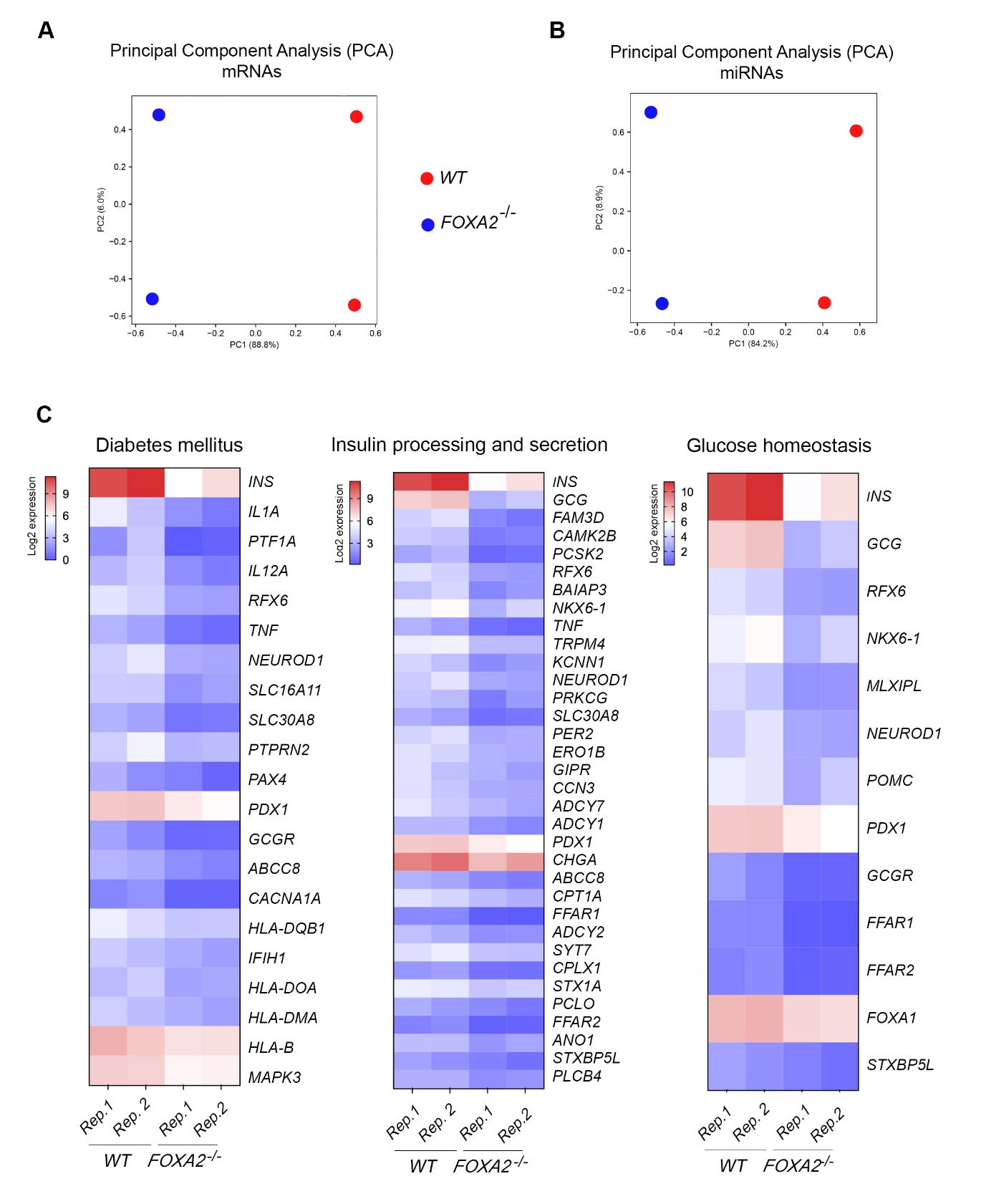


**Supplementary Figure 1.** **Enriched signaling pathways of downregulated DEGs in *FOXA2****^–/–^* **islets.** Principal component analyses (PCA) showing clear separation between DEGs **(A)** and DEmiRs **(B)** from *FOXA2^–/–^* islets and WT-islets. **(C)** Heatmaps of downregulated DEGs associated with diabetes mellitus, insulin processing, insulin secretion, and glucose homeostasis in FOXA2 absence using KEGG pathway and DAVID Tool.
