# Supplementary material for: Identifying miRNA Signatures Associated with Pancreatic Islet Dysfunction in a FOXA2-Deficient iPSC Model
Source: Stem Cell Rev Rep. 2024 Jun 25;20(7):1915–31. doi: 10.1007/s12015-024-10752-0 (PMC11445299; doi:10.1007/s12015-024-10752-0)
Supplement: Supplementary file 2 — Supplementary Material 2 [file 12015_2024_10752_MOESM2_ESM.docx]

**Supplementary Table 2.** List of primers and miRNA assays used for RT-qPCR validation.

| **List of primers** | | |
| --- | --- | --- |
| **Gene** | **Forward** | **Reverse** |
| *INS* | AAGAGGCCATCAAGCAGATCA | CAGGAGGCGCATCCACA |
| *GCG* | CTCTTCACCTGCTCTGTTCTAC | TGGATTTCTCCTCTGTGTCTTG |
| *PPY* | AGGTGCTCGCTTGGTCTAGTG | ACCCAGCAGTGGCTGTAGTAAC |
| *IAPP* | TTGAGAAGCAATGGGCATCC | GGGTGTAGCTTTCAGATGGTTC |
| *ARX* | CTGCTGAAACGCAAACAGAGGC | CTCGGTCAAGTCCAGCCTCATG |
| *NKX6.1* | GGGCTCGTTTGGCCTATTCGTT | CCACTTGGTCCGGCGGTTCT |
| *PDX1* | CGTCCAGCTGCCTTTCCCAT | CCGTGAGATGTACTTGTTGAATAGGA |
| *GCGR* | AGGTGATGGACTTCCTGTTTGAG | TACTTGTCGAAGGTTCTGTTGC |
| *UCN3* | GATGGGCTTGGCTTTGTAGA | GGAGGGAAGTCCACTCTCG |
| *NEUROG3* | GGCTGTGGGTGCTAAGGGTAAG | CAGGGAGAAGCAGAAGGAACAA |
| *NEUROD1* | GCCCCAGGGTTATGAGACTAT | GAGAACTGAGACACTCGTCTGT |
| *NKX2.2* | AAACCATGTCACGCGCTCA | GGCGTTGTACTGCATGTGCT |
| *INSM1* | TTTGTCTCGTGGTTGGAAGC | CCAAAACAACCCGTACGCTA |
| *PAX4* | AGCAGAGGCACTGGAGAAAGAGTT | CAGCTGCATTTCCCACTTGAGCTT |
| *PAX6* | GCGGAAGCTGCAAAGAAATAG | GGGCAAACACATCTGGATAATG |
| *RFX6* | GTCGATGCATGGCTTGGACT | TGGGCCATAGCTAGACGGTG |
| *HES1* | AGTGAAGCACCTCCGGAAC | TCACCTCGTTCATGCACTC |
| *HES6* | AGCCCCTGGTGGAGAAGA | CAGCACTCCGGCGTTCTC |
| *PTF1A* | CCAGAAGGTCATCATCTGCC | AGAGAGTGTCCTGCTAGGGG |
| *ABCC6* | TGGATCATAGTGCTGGCAAATG | GTTTCTCCTTCTCCTCTATCTCC |
| *AFP* | ACAGAGGAACAACTTGAGGCTGTC | AGCAAAGCAGACTTCCTGTTCCTG |
| *APOA2* | GTTCGGAGACAGGCAAAGGA | TCAAAGTAAGACTTGGCCTCGG |
| *APOC3* | CTTCATGCAGGGTTACATGAAG | TTTCAGGGAACTGAAGCCATC |
| *HHEX* | GCGAGAGACAGGTCAAAACC | AGGGCGAACATTGAGAGCTA |
| *LIN28A* | GGTGCGGGCATCTGTAAGTG | GGAACCCTTCCATGTGCAGC |
| *NPY* | CGCTGCGACACTACATCAACC | AGGGTCTTCAAGCCGAGTTCTG |
| *OLIG3* | GCGAGAGAGATCAAGACCCA | ACTGCGTCGAAAGGAAAACC |
| *SST* | AGCTGCTGTCTGAACCCAAC | CCATAGCCGGGTTTGAGTTA |
| *GAPDH* | ACGACCACTTTGTCAAGCTCATTTC | GCAGTGAGGGTCTCTCTCTTCCTCT |

| **miRNA Assays** | |
| --- | --- |
| **miRNA PCR Assay** | **Assay ID** |
| hsa-miR-199a-5p | YP00204494 |
| hsa-miR-199a-3p | YP00204536 |
| hsa-miR-204-5p | YP00206072 |
| hsa-miR-1-3p | YP00204344 |
| hsa-miR-383-5p | YP00205904 |
| hsa-miR-10b-5p | YP00205637 |
| hsa-miR-218-5p | YP00206034 |
| hsa-miR-137-3p | YP00206062 |
| hsa-miR-155-5p | YP02119311 |
| hsa-miR-105-5p | YP00204389 |
| hsa-miR-337-3p | YP00205938 |
| hsa-miR-542-3p | YP00205444 |
| hsa-miR-340-5p | YP00206068 |
| hsa-miR-203a-3p | YP00205914 |
| hsa-miR-99a-3p | YP00204523 |
| hsa-miR-409-3p | YP00204358 |
| hsa-miR-934 | YP02119292 |
| hsa-miR-136-3p | YP00205503 |
| hsa-let-7f-2-3p | YP00204095 |
| hsa-miR-203b-3p | YP02103829 |
| hsa-miR-98-5p | YP00204640 |
| hsa-miR-493-3p | YP00204557 |
| hsa-miR-122-5p | YP00205664 |
| hsa-miR-371a-3p | YP00204299 |
| hsa-miR-371a-5p | YP00204493 |
| hsa-miR-373-3p | YP00204604 |
| SNORD48(hsa) | YP00203903 |
