# Supplementary material for: Identifying miRNA Signatures Associated with Pancreatic Islet Dysfunction in a FOXA2-Deficient iPSC Model
Source: Stem Cell Rev Rep. 2024 Jun 25;20(7):1915–31. doi: 10.1007/s12015-024-10752-0 (PMC11445299; doi:10.1007/s12015-024-10752-0)
Supplement: Supplementary file 3 — Supplementary Material 3 [file 12015_2024_10752_MOESM3_ESM.docx]

**Supplementary Table 3.** Top upregulated DEGs in *FOXA2^–/–^* islets compared with WT-islets (Log2 FC > 1, *P* < 0.05).

| **Gene ID** | **Log2 FC** | ***P*-value** |
| --- | --- | --- |
| *KRT1* | 5.883013 | 0.000985 |
| *COL3A1* | 5.782581 | 0.000195 |
| *CALB1* | 5.479372 | 0.000017 |
| *APOA2* | 5.433256 | 0.000005 |
| *A2M* | 5.266395 | 0.009813 |
| *RELN* | 5.035494 | 0.000034 |
| *SOX17* | 4.794410 | 0.000411 |
| *COL1A2* | 4.736296 | 0.000725 |
| *FGG* | 4.704527 | 0.000107 |
| *AFP* | 4.526164 | 0.000698 |
| *GABRP* | 4.445883 | 0.000942 |
| *PTX3* | 4.402107 | 0.000166 |
| *CXCL14* | 4.340724 | 0.019216 |
| *APOA4* | 4.278651 | 0.014152 |
| *POSTN* | 4.219276 | 0.000294 |
| *HSD3B1* | 4.138513 | 0.002163 |
| *SPARCL1* | 4.125078 | 0.000231 |
| *WNT2B* | 4.066964 | 0.000359 |
| *LIN28A* | 4.004317 | 0.000051 |
| *ACTA2* | 3.986646 | 0.003101 |
| *CD248* | 3.980332 | 0.000324 |
| *ADGRA2* | 3.913052 | 0.000089 |
| *APOC3* | 3.897227 | 0.006166 |
| *MYBPC3* | 3.847898 | 0.000024 |
| *AHSG* | 3.843007 | 0.000050 |
| *KCNJ8* | 3.741418 | 0.000881 |
| *C6* | 3.725187 | 0.000042 |
| *RBP4* | 3.724246 | 0.000873 |
| *COL11A1* | 3.710116 | 0.000227 |
| *ACTC1* | 3.669441 | 0.002052 |
| *BGN* | 3.665861 | 0.001290 |
| *ALKAL2* | 3.619271 | 0.000041 |
| *HAND1* | 3.618144 | 0.007086 |
| *NPY* | 3.603045 | 0.001557 |
| *GATA5* | 3.581672 | 0.000418 |
| *GPC3* | 3.570344 | 0.001960 |
| *DUSP9* | 3.558037 | 0.000126 |
| *BMP4* | 3.557951 | 0.000201 |
| *COL9A3* | 3.519734 | 0.000177 |
| *LCN15* | 3.506077 | 0.002915 |
| *DES* | 3.505785 | 0.002163 |
| *CDKN1C* | 3.481887 | 0.000074 |
| *DCN* | 3.477796 | 0.002240 |
| *CLMP* | 3.463822 | 0.000077 |
| *VAT1L* | 3.450420 | 0.000271 |
| *OLIG3* | 3.438869 | 0.000910 |
| *CXCL12* | 3.427706 | 0.003097 |
| *COL6A3* | 3.366678 | 0.002987 |
| *URAD* | 3.360436 | 0.000193 |
| *TTYH1* | 3.323021 | 0.001042 |
| *ASGR2* | 3.297619 | 0.000044 |
| *HTRA1* | 3.291037 | 0.007451 |
| *F2* | 3.257104 | 0.004818 |
| *WNT6* | 3.256271 | 0.010484 |
| *PITX2* | 3.241344 | 0.000034 |
| *SFRP5* | 3.205634 | 0.003585 |
| *FGB* | 3.147667 | 0.033733 |
| *SEMA6B* | 3.141794 | 0.000401 |
| *COL5A2* | 3.140943 | 0.000436 |
| *TGFBI* | 3.119701 | 0.006841 |
| *LGR5* | 3.099138 | 0.000449 |
| *GDF6* | 3.087368 | 0.000591 |
| *SRGN* | 3.075938 | 0.001392 |
| *PTN* | 3.070695 | 0.000677 |
| *EDNRB* | 3.070232 | 0.000141 |
| *PTH1R* | 3.041565 | 0.000053 |
| *PDGFRB* | 3.034967 | 0.000385 |
| *HAPLN1* | 3.029612 | 0.000470 |
| *KRTDAP* | 3.023072 | 0.043904 |
| *GAP43* | 3.021519 | 0.020357 |
| *ZNF521* | 3.010242 | 0.000458 |
| *DSG1* | 3.006862 | 0.003137 |
| *PDGFRA* | 2.958354 | 0.000049 |
| *MYL7* | 2.952391 | 0.009990 |
| *GLDC* | 2.927665 | 0.000490 |
| *GABRA2* | 2.926765 | 0.000792 |
| *HHIP* | 2.916994 | 0.000907 |
| *LGALS14* | 2.900540 | 0.033138 |
| *HMGCS2* | 2.891864 | 0.001677 |
| *UGT2B11* | 2.879645 | 0.007328 |
| *FGA* | 2.869400 | 0.003365 |
| *TFAP2B* | 2.867028 | 0.002359 |
| *B3GALT1* | 2.864150 | 0.000771 |
| *NTRK2* | 2.860824 | 0.007615 |
| *OCA2* | 2.857906 | 0.000306 |
| *EMILIN1* | 2.854771 | 0.017253 |
| *CTNNA2* | 2.852362 | 0.000295 |
| *ANPEP* | 2.850059 | 0.001980 |
| *HOXB2* | 2.841496 | 0.008615 |
| *PCSK5* | 2.826800 | 0.000312 |
| *AMBP* | 2.817976 | 0.026671 |
| *PENK* | 2.814065 | 0.004658 |
| *APOE* | 2.811868 | 0.001774 |
| *GATA2* | 2.806731 | 0.001192 |
| *SLC22A10* | 2.804778 | 0.009672 |
| *ERVV-1* | 2.802207 | 0.000203 |
| *CRABP1* | 2.798465 | 0.049272 |
| *APCS* | 2.796480 | 0.001975 |
| *HSPB6* | 2.795951 | 0.001635 |
| *HAND2* | 2.788982 | 0.008251 |
| *IGFBP3* | 2.783111 | 0.002383 |
| *PLA2G2A* | 2.781383 | 0.012291 |
| *CA4* | 2.776809 | 0.012719 |
| *GAS1* | 2.775482 | 0.015586 |
| *FZD4* | 2.772512 | 0.000062 |
| *COL9A2* | 2.746627 | 0.000854 |
| *ADCYAP1* | 2.725630 | 0.000704 |
| *TMEM130* | 2.711961 | 0.004430 |
| *CAPN6* | 2.694053 | 0.000100 |
| *ACTG2* | 2.691711 | 0.008602 |
| *SFRP1* | 2.668163 | 0.000147 |
| *PCP4* | 2.650810 | 0.000294 |
| *DDR2* | 2.638976 | 0.000618 |
| *DPP4* | 2.635987 | 0.023592 |
| *TBX2* | 2.635329 | 0.001674 |
| *CNN1* | 2.632396 | 0.001149 |
| *TRABD2B* | 2.629211 | 0.000479 |
| *LOXL2* | 2.611407 | 0.000978 |
| *DAAM2* | 2.610841 | 0.001722 |
| *LOX* | 2.608137 | 0.000210 |
| *MRC2* | 2.598063 | 0.002786 |
| *NRP2* | 2.595536 | 0.004361 |
| *TSPAN18* | 2.595432 | 0.000093 |
| *TPM2* | 2.593790 | 0.001563 |
| *ROR1* | 2.588043 | 0.000290 |
| *CST1* | 2.577324 | 0.000219 |
| *APOB* | 2.569907 | 0.031380 |
| *LIN7A* | 2.567268 | 0.000076 |
| *NKX1-2* | 2.564435 | 0.002282 |
| *CNTFR* | 2.555445 | 0.019535 |
| *APOC2* | 2.534667 | 0.008866 |
| *PDZK1* | 2.521182 | 0.019898 |
| *SPSB4* | 2.521021 | 0.000595 |
| *SLC19A3* | 2.520547 | 0.005894 |
| *LIPC* | 2.506663 | 0.002433 |
| *MYL4* | 2.504500 | 0.019959 |
| *SLC17A4* | 2.504090 | 0.005968 |
| *COL8A1* | 2.497091 | 0.000683 |
| *CPA2* | 2.492019 | 0.003022 |
| *CRB2* | 2.489605 | 0.000451 |
| *SBSPON* | 2.487491 | 0.003741 |
| *TNC* | 2.476923 | 0.000286 |
| *SALL3* | 2.471260 | 0.000098 |
| *KCNIP1* | 2.466084 | 0.009811 |
| *PRSS35* | 2.461024 | 0.000100 |
| *TSPAN8* | 2.441167 | 0.002133 |
| *TTR* | 2.434215 | 0.004597 |
| *PCDH17* | 2.430816 | 0.000176 |
| *MATN2* | 2.429981 | 0.000298 |
| *APOA1* | 2.415987 | 0.004667 |
| *MMP24* | 2.408768 | 0.000790 |
| *NEFM* | 2.407282 | 0.000774 |
| *FGF2* | 2.402290 | 0.000148 |
| *ENG* | 2.399158 | 0.000093 |
| *PKDCC* | 2.389005 | 0.005266 |
| *SCG2* | 2.388477 | 0.002145 |
| *CTHRC1* | 2.380752 | 0.006882 |
| *SELENOP* | 2.379562 | 0.000788 |
| *SERPINA5* | 2.376276 | 0.002514 |
| *DACT1* | 2.366551 | 0.003592 |
| *IGDCC3* | 2.360696 | 0.002721 |
| *SHOX2* | 2.352232 | 0.009379 |
| *ENPP7* | 2.347244 | 0.035730 |
| *OLFML3* | 2.346314 | 0.002786 |
| *NPR3* | 2.342991 | 0.007742 |
| *HMOX1* | 2.339864 | 0.002408 |
| *MTTP* | 2.339240 | 0.011627 |
| *TIMP3* | 2.333180 | 0.001451 |
| *CD244* | 2.320870 | 0.031767 |
| *LRRTM1* | 2.317746 | 0.005312 |
| *EDN3* | 2.314412 | 0.021595 |
| *NPTX2* | 2.287396 | 0.006995 |
| *IGLON5* | 2.287204 | 0.006647 |
| *PMP22* | 2.283389 | 0.002400 |
| *NEFL* | 2.282019 | 0.004551 |
| *HOXB4* | 2.274054 | 0.002251 |
| *CLU* | 2.273067 | 0.006682 |
| *CST4* | 2.272256 | 0.000108 |
| *CYTL1* | 2.262476 | 0.015040 |
| *UGT2A3* | 2.252964 | 0.016734 |
| *PAH* | 2.238536 | 0.020612 |
| *PF4* | 2.237150 | 0.001896 |
| *TF* | 2.230508 | 0.001212 |
| *ELOVL2* | 2.225563 | 0.002885 |
| *EDNRA* | 2.220325 | 0.001145 |
| *RGS5* | 2.219339 | 0.001927 |
| *CLDN2* | 2.211100 | 0.004140 |
| *FBN1* | 2.209027 | 0.000430 |
| *BTNL3* | 2.202721 | 0.005767 |
| *NR2F2* | 2.198754 | 0.000651 |
| *CUX2* | 2.193826 | 0.042671 |
| *NTNG1* | 2.191506 | 0.000599 |
| *PREX1* | 2.191205 | 0.025568 |
| *FAM151A* | 2.188458 | 0.001172 |
| *SDK2* | 2.178335 | 0.001066 |
| *ITGA11* | 2.174331 | 0.002998 |
| *HOGA1* | 2.168276 | 0.002259 |
| *FLRT2* | 2.164832 | 0.046475 |
| *BAMBI* | 2.159477 | 0.000207 |
| *MMP2* | 2.157184 | 0.016318 |
| *LZTS1* | 2.147587 | 0.003166 |
| *SAMD11* | 2.147429 | 0.005941 |
| *DSC3* | 2.144961 | 0.001131 |
| *DKK2* | 2.140417 | 0.009316 |
| *LRRN2* | 2.139285 | 0.000134 |
| *WNT9B* | 2.130923 | 0.001330 |
| *SLIT2* | 2.123351 | 0.000898 |
| *CTSC* | 2.114790 | 0.000272 |
| *SDC2* | 2.111606 | 0.014508 |
| *LAMA2* | 2.102461 | 0.000191 |
| *ANXA13* | 2.098008 | 0.004191 |
| *TUBB4A* | 2.097017 | 0.008472 |
| *FGF19* | 2.086062 | 0.026446 |
| *LUM* | 2.085002 | 0.011922 |
| *FST* | 2.082178 | 0.049763 |
| *FBLN5* | 2.076190 | 0.012916 |
| *PHYHIPL* | 2.065911 | 0.003713 |
| *PCDHGB7* | 2.061351 | 0.002564 |
| *TRH* | 2.061298 | 0.000752 |
| *BEX1* | 2.061178 | 0.003811 |
| *AKR1D1* | 2.047309 | 0.021353 |
| *MSN* | 2.046045 | 0.009079 |
| *PRTG* | 2.040664 | 0.007605 |
| *APCDD1* | 2.031217 | 0.014344 |
| *COL1A1* | 2.020142 | 0.006933 |
| *MGP* | 2.010206 | 0.031444 |
| *ADAMTS12* | 2.006529 | 0.000522 |
| *MMP16* | 2.004701 | 0.017031 |
| *SLC47A1* | 1.995437 | 0.000297 |
| *SLIT3* | 1.992052 | 0.000376 |
| *BAAT* | 1.987333 | 0.001823 |
| *NLGN4X* | 1.985241 | 0.000748 |
| *DACT2* | 1.981547 | 0.000480 |
| *TLX3* | 1.980741 | 0.000286 |
| *MAN1C1* | 1.979755 | 0.000433 |
| *NID2* | 1.961793 | 0.000978 |
| *GASK1B* | 1.961490 | 0.002402 |
| *SOAT2* | 1.950445 | 0.000241 |
| *LGALS2* | 1.942708 | 0.014338 |
| *UCHL1* | 1.941570 | 0.011738 |
| *SYDE1* | 1.940255 | 0.009649 |
| *ALPI* | 1.938137 | 0.022791 |
| *MCAM* | 1.938045 | 0.000854 |
| *MYH6* | 1.937293 | 0.013701 |
| *GUCY2C* | 1.936311 | 0.012999 |
| *RHOJ* | 1.931813 | 0.000334 |
| *PDGFD* | 1.928530 | 0.000457 |
| *SYNPO2* | 1.927985 | 0.000906 |
| *GDF7* | 1.927483 | 0.001684 |
| *SLCO2B1* | 1.913000 | 0.005740 |
| *TBX18* | 1.910745 | 0.002291 |
| *CNTN6* | 1.906387 | 0.000490 |
| *L1CAM* | 1.901132 | 0.007481 |
| *DPEP1* | 1.899975 | 0.005106 |
| *ACTN3* | 1.892337 | 0.003327 |
| *COL2A1* | 1.884383 | 0.000490 |
| *DCHS2* | 1.879436 | 0.013558 |
| *NRK* | 1.877832 | 0.002455 |
| *KANK4* | 1.877680 | 0.011867 |
| *C7* | 1.873867 | 0.001195 |
| *LRRC32* | 1.863560 | 0.000418 |
| *SEMA5A* | 1.859240 | 0.007233 |
| *EGF* | 1.858628 | 0.002687 |
| *CTSF* | 1.856693 | 0.000620 |
| *ST6GAL2* | 1.851025 | 0.025164 |
| *FHL1* | 1.847895 | 0.034701 |
| *HOXB3* | 1.846972 | 0.003910 |
| *TENM3* | 1.846714 | 0.000394 |
| *LEFTY1* | 1.840215 | 0.001248 |
| *FAM20A* | 1.838669 | 0.026073 |
| *WNT5A* | 1.836846 | 0.011571 |
| *PAGE4* | 1.833917 | 0.027329 |
| *WSCD2* | 1.830264 | 0.027807 |
| *SLC5A9* | 1.827910 | 0.010260 |
| *ALDH1A1* | 1.827323 | 0.001934 |
| *CA14* | 1.825735 | 0.006133 |
| *DCDC2* | 1.825383 | 0.001056 |
| *KITLG* | 1.822008 | 0.003114 |
| *FABP1* | 1.817167 | 0.009621 |
| *AQP10* | 1.815680 | 0.004675 |
| *TFEC* | 1.814297 | 0.028170 |
| *NAALAD2* | 1.814110 | 0.000846 |
| *PLAT* | 1.813647 | 0.003309 |
| *ORM1* | 1.805846 | 0.005113 |
| *HEG1* | 1.799395 | 0.033885 |
| *BNC1* | 1.797216 | 0.001933 |
| *SOSTDC1* | 1.795888 | 0.020262 |
| *MSX2* | 1.791604 | 0.013408 |
| *KCNQ1* | 1.791418 | 0.009810 |
| *LEFTY2* | 1.786042 | 0.000464 |
| *RSPO3* | 1.781372 | 0.016298 |
| *PTGIS* | 1.776587 | 0.000727 |
| *L1TD1* | 1.772977 | 0.000378 |
| *CSPG4* | 1.766219 | 0.009690 |
| *CPB2* | 1.761378 | 0.010662 |
| *VIM* | 1.761021 | 0.002130 |
| *OAF* | 1.757908 | 0.002238 |
| *GNG11* | 1.755994 | 0.032527 |
| *DAB2* | 1.754970 | 0.002584 |
| *NR5A2* | 1.753504 | 0.005532 |
| *GCNT2* | 1.752831 | 0.000640 |
| *SERPIND1* | 1.747175 | 0.001360 |
| *CPA4* | 1.743376 | 0.008392 |
| *GP6* | 1.740278 | 0.004107 |
| *ZEB1* | 1.727942 | 0.003952 |
| *ARHGAP24* | 1.724181 | 0.003787 |
| *TRIL* | 1.723680 | 0.014877 |
| *GABRB1* | 1.718909 | 0.007139 |
| *FXYD2* | 1.716178 | 0.008968 |
| *CA12* | 1.711974 | 0.012757 |
| *SLC1A1* | 1.705720 | 0.044881 |
| *CST5* | 1.705703 | 0.018085 |
| *UNC5CL* | 1.703325 | 0.033826 |
| *SLC26A2* | 1.701462 | 0.003734 |
| *IRX4* | 1.700900 | 0.035185 |
| *STRA6* | 1.700110 | 0.002655 |
| *ROR2* | 1.696830 | 0.000455 |
| *ANO4* | 1.696258 | 0.000379 |
| *SYNC* | 1.694059 | 0.004235 |
| *B3GALT5* | 1.693801 | 0.004749 |
| *GATA3* | 1.693652 | 0.022217 |
| *LGI2* | 1.692972 | 0.041934 |
| *TMEM72* | 1.686230 | 0.000849 |
| *SLC35G1* | 1.686037 | 0.008134 |
| *PHOX2A* | 1.681287 | 0.008623 |
| *HTR1E* | 1.676899 | 0.000377 |
| *HSPB7* | 1.671943 | 0.019011 |
| *TNNT2* | 1.671525 | 0.002642 |
| *IRX2* | 1.669588 | 0.046334 |
| *MRAP2* | 1.659529 | 0.016440 |
| *ITGA9* | 1.657913 | 0.004900 |
| *ADCY8* | 1.646068 | 0.023116 |
| *CDH13* | 1.642811 | 0.022391 |
| *GRIK3* | 1.640550 | 0.000533 |
| *SULT1B1* | 1.639036 | 0.015702 |
| *CKMT2* | 1.633567 | 0.001144 |
| *LEF1* | 1.629145 | 0.012260 |
| *APBB1IP* | 1.625831 | 0.001241 |
| *SMLR1* | 1.625366 | 0.024146 |
| *CRYBB3* | 1.623460 | 0.021281 |
| *CRYAB* | 1.622985 | 0.040302 |
| *ACTA1* | 1.615135 | 0.008079 |
| *ENOX1* | 1.612433 | 0.003838 |
| *NEXN* | 1.612368 | 0.007263 |
| *ART5* | 1.610906 | 0.008203 |
| *SOBP* | 1.610676 | 0.002787 |
| *NCAM1* | 1.610379 | 0.000866 |
| *F2RL2* | 1.610100 | 0.000912 |
| *JPH2* | 1.609423 | 0.008151 |
| *ODAM* | 1.604262 | 0.034433 |
| *PPP1R14A* | 1.603590 | 0.034326 |
| *PCDH10* | 1.597152 | 0.005040 |
| *ASPHD1* | 1.590437 | 0.004026 |
| *LSAMP* | 1.587323 | 0.007760 |
| *ANGPTL2* | 1.581766 | 0.007601 |
| *SLITRK4* | 1.579966 | 0.000515 |
| *PLA2G12B* | 1.577241 | 0.020816 |
| *GPR50* | 1.575031 | 0.000436 |
| *LMOD1* | 1.574687 | 0.002533 |
| *GRIN2A* | 1.573834 | 0.001119 |
| *IGFL2* | 1.571905 | 0.028467 |
| *PLPPR3* | 1.567840 | 0.008860 |
| *TMEM86B* | 1.567016 | 0.013081 |
| *TGFB2* | 1.561504 | 0.046679 |
| *SVEP1* | 1.560967 | 0.003064 |
| *CIDEC* | 1.559138 | 0.005503 |
| *MPV17L* | 1.558029 | 0.001749 |
| *CBLN2* | 1.558007 | 0.001284 |
| *MSRB3* | 1.550480 | 0.002755 |
| *GSTA2* | 1.547909 | 0.015894 |
| *FAM89A* | 1.547236 | 0.015393 |
| *ST3GAL5* | 1.546650 | 0.006655 |
| *TMEM37* | 1.545770 | 0.002387 |
| *PRAP1* | 1.545296 | 0.035490 |
| *MEDAG* | 1.544425 | 0.047564 |
| *MLN* | 1.543389 | 0.013358 |
| *ANXA6* | 1.542229 | 0.019519 |
| *ERVV-2* | 1.539897 | 0.012614 |
| *SLC22A8* | 1.536774 | 0.000518 |
| *RASSF5* | 1.536760 | 0.001798 |
| *GPR37* | 1.536382 | 0.001365 |
| *C8orf88* | 1.535774 | 0.041955 |
| *MRGPRF* | 1.535746 | 0.027817 |
| *SERPINA6* | 1.535530 | 0.001804 |
| *SST* | 1.532785 | 0.013788 |
| *LRRTM4* | 1.531449 | 0.001768 |
| *GC* | 1.527058 | 0.001295 |
| *GAL3ST3* | 1.526524 | 0.007559 |
| *ARSI* | 1.519843 | 0.001764 |
| *PLCXD3* | 1.516728 | 0.001865 |
| *DKK1* | 1.516249 | 0.002554 |
| *CCND2* | 1.513780 | 0.029444 |
| *TMEM132D* | 1.513040 | 0.000723 |
| *CCDC3* | 1.510894 | 0.016466 |
| *VCAN* | 1.510883 | 0.027490 |
| *BCHE* | 1.503736 | 0.024205 |
| *SNAI1* | 1.501577 | 0.011854 |
| *MME* | 1.499770 | 0.008825 |
| *UGT2B7* | 1.498604 | 0.035247 |
| *BMP5* | 1.494263 | 0.009161 |
| *CST2* | 1.493122 | 0.014870 |
| *GGT5* | 1.491470 | 0.015580 |
| *MAB21L2* | 1.488758 | 0.026530 |
| *TMCC3* | 1.487999 | 0.001294 |
| *FAM13C* | 1.486501 | 0.002018 |
| *SMPX* | 1.483645 | 0.015497 |
| *CYP27A1* | 1.482929 | 0.002474 |
| *NGFR* | 1.480225 | 0.004048 |
| *AFF3* | 1.478310 | 0.002390 |
| *VEPH1* | 1.477019 | 0.011578 |
| *FIGNL2* | 1.476781 | 0.007346 |
| *SOX9* | 1.474219 | 0.025428 |
| *SLC39A14* | 1.471627 | 0.003748 |
| *MN1* | 1.470074 | 0.003879 |
| *NRXN3* | 1.463286 | 0.002595 |
| *BMERB1* | 1.462994 | 0.002466 |
| *EPDR1* | 1.462623 | 0.001620 |
| *DLC1* | 1.457579 | 0.002835 |
| *CNMD* | 1.450556 | 0.026732 |
| *VIP* | 1.449980 | 0.029939 |
| *PCOLCE* | 1.447663 | 0.019370 |
| *BICC1* | 1.447600 | 0.001326 |
| *LAMB1* | 1.443973 | 0.014131 |
| *SCD* | 1.439980 | 0.003656 |
| *SNAI2* | 1.439954 | 0.015293 |
| *C8B* | 1.438702 | 0.005437 |
| *DEPDC7* | 1.438655 | 0.002042 |
| *KDR* | 1.435198 | 0.000756 |
| *RNF175* | 1.432464 | 0.011432 |
| *SLC38A4* | 1.430860 | 0.035050 |
| *ORM2* | 1.430573 | 0.006692 |
| *DAB1* | 1.428542 | 0.000844 |
| *COL4A6* | 1.426642 | 0.003521 |
| *TNFRSF19* | 1.425169 | 0.002162 |
| *HRK* | 1.421023 | 0.002131 |
| *GSTM4* | 1.419109 | 0.000898 |
| *CD3D* | 1.418398 | 0.037139 |
| *TENM2* | 1.417099 | 0.000799 |
| *TUBB8B* | 1.416827 | 0.001046 |
| *ESM1* | 1.416149 | 0.002093 |
| *EBF3* | 1.415937 | 0.002777 |
| *THBS2* | 1.412757 | 0.037397 |
| *ISL1* | 1.410521 | 0.024911 |
| *TNMD* | 1.407704 | 0.001462 |
| *TMEM178A* | 1.406760 | 0.002389 |
| *ZCCHC24* | 1.406001 | 0.006648 |
| *REC8* | 1.405521 | 0.017390 |
| *GLT8D2* | 1.403017 | 0.047838 |
| *TENM1* | 1.399052 | 0.005447 |
| *ZFPM2* | 1.397902 | 0.000837 |
| *TCEAL7* | 1.397624 | 0.002762 |
| *AQP1* | 1.396664 | 0.002310 |
| *CYP26A1* | 1.395342 | 0.042842 |
| *C21orf62* | 1.394456 | 0.040414 |
| *EDIL3* | 1.382223 | 0.014265 |
| *STARD8* | 1.380332 | 0.000924 |
| *AJAP1* | 1.379777 | 0.001106 |
| *HMCN1* | 1.379167 | 0.006607 |
| *HSD17B2* | 1.377997 | 0.004636 |
| *HOXA3* | 1.374232 | 0.000813 |
| *DACH1* | 1.373662 | 0.002949 |
| *GLTPD2* | 1.373597 | 0.005440 |
| *CSRP2* | 1.370522 | 0.005133 |
| *CPQ* | 1.369648 | 0.013889 |
| *SLC51B* | 1.368727 | 0.004343 |
| *HCN4* | 1.367373 | 0.006281 |
| *SCARB1* | 1.366789 | 0.007276 |
| *THY1* | 1.365312 | 0.040795 |
| *SLC18A3* | 1.361222 | 0.005370 |
| *GRK3* | 1.360033 | 0.008625 |
| *LRFN1* | 1.356770 | 0.002687 |
| *NDST3* | 1.353193 | 0.005164 |
| *SLC16A10* | 1.352834 | 0.001920 |
| *NELL1* | 1.351754 | 0.008777 |
| *CCDC160* | 1.351746 | 0.024057 |
| *FJX1* | 1.350620 | 0.044612 |
| *WIF1* | 1.348229 | 0.003484 |
| *CCBE1* | 1.346778 | 0.013889 |
| *NDP* | 1.346029 | 0.014030 |
| *SLN* | 1.341861 | 0.001724 |
| *ITLN2* | 1.340144 | 0.018575 |
| *KCNIP4* | 1.338315 | 0.004112 |
| *COL4A1* | 1.337453 | 0.008139 |
| *PCYT1B* | 1.335926 | 0.003960 |
| *KIF26B* | 1.334998 | 0.006668 |
| *CRTAC1* | 1.334596 | 0.004864 |
| *GGTLC2* | 1.331536 | 0.020216 |
| *F7* | 1.329207 | 0.002147 |
| *FADS6* | 1.327434 | 0.005543 |
| *ARSL* | 1.322927 | 0.014098 |
| *CCDC88A* | 1.319947 | 0.001397 |
| *MGARP* | 1.318127 | 0.014296 |
| *IGFBP7* | 1.314383 | 0.006204 |
| *DPYSL4* | 1.312680 | 0.005336 |
| *MMP23B* | 1.309773 | 0.017805 |
| *C9orf64* | 1.309456 | 0.003274 |
| *TRPV4* | 1.307315 | 0.023423 |
| *ENPEP* | 1.304863 | 0.002700 |
| *SERPINB9* | 1.302230 | 0.004443 |
| *PEAR1* | 1.298009 | 0.001352 |
| *SORCS3* | 1.294748 | 0.009826 |
| *TMEM190* | 1.292417 | 0.002577 |
| *ETV1* | 1.290868 | 0.002758 |
| *FGF9* | 1.289809 | 0.005111 |
| *SCUBE3* | 1.288436 | 0.035331 |
| *WNT11* | 1.287608 | 0.013312 |
| *MFAP4* | 1.285477 | 0.005363 |
| *CACHD1* | 1.283157 | 0.002487 |
| *ITGA5* | 1.282483 | 0.025046 |
| *CNR1* | 1.282374 | 0.008660 |
| *BTNL8* | 1.280573 | 0.004640 |
| *SPHK1* | 1.277174 | 0.006250 |
| *PKNOX2* | 1.270282 | 0.010115 |
| *DDIT4L* | 1.268495 | 0.011158 |
| *TRPM6* | 1.267600 | 0.001635 |
| *ANXA9* | 1.266715 | 0.038345 |
| *BDNF* | 1.266096 | 0.042257 |
| *STXBP6* | 1.264670 | 0.004667 |
| *OGDHL* | 1.259711 | 0.001553 |
| *LAMA4* | 1.258547 | 0.007160 |
| *METTL24* | 1.257498 | 0.001946 |
| *FGFR4* | 1.255439 | 0.048591 |
| *CASR* | 1.254626 | 0.030478 |
| *NPFFR2* | 1.254124 | 0.007656 |
| *SRPX* | 1.253947 | 0.003574 |
| *SHC3* | 1.252854 | 0.006695 |
| *BNC2* | 1.252284 | 0.004811 |
| *ID2* | 1.251890 | 0.001499 |
| *SULF1* | 1.250796 | 0.024674 |
| *ITGA8* | 1.248090 | 0.001082 |
| *IL1RAPL1* | 1.246909 | 0.030676 |
| *LDLRAD3* | 1.243640 | 0.003929 |
| *GNB4* | 1.242735 | 0.015706 |
| *P2RY6* | 1.241120 | 0.002476 |
| *ENPP1* | 1.238236 | 0.003533 |
| *C1QL2* | 1.237982 | 0.003077 |
| *ALKAL1* | 1.237043 | 0.024648 |
| *SFMBT2* | 1.235819 | 0.005136 |
| *COL21A1* | 1.233505 | 0.031903 |
| *COL4A2* | 1.233436 | 0.003361 |
| *RASGRF2* | 1.233187 | 0.007701 |
| *RALYL* | 1.232924 | 0.004688 |
| *HSD17B11* | 1.231445 | 0.023952 |
| *BACH2* | 1.231303 | 0.008818 |
| *NOX1* | 1.230367 | 0.017246 |
| *FZD8* | 1.228044 | 0.001372 |
| *SLC17A1* | 1.225831 | 0.021320 |
| *MYLK* | 1.225777 | 0.002820 |
| *FBXL7* | 1.225636 | 0.002627 |
| *FLT1* | 1.222638 | 0.041628 |
| *TNFSF4* | 1.222247 | 0.004209 |
| *PLTP* | 1.221591 | 0.002654 |
| *PTGER3* | 1.220634 | 0.031494 |
| *CHST13* | 1.219168 | 0.013457 |
| *TMEM200B* | 1.218870 | 0.006916 |
| *IQGAP2* | 1.218573 | 0.003175 |
| *PECAM1* | 1.213343 | 0.004343 |
| *CYP3A7* | 1.209276 | 0.047796 |
| *VSNL1* | 1.208692 | 0.004867 |
| *RGN* | 1.204175 | 0.011920 |
| *EPB41L2* | 1.203602 | 0.003557 |
| *PIEZO2* | 1.201767 | 0.025121 |
| *NR0B2* | 1.199482 | 0.004540 |
| *GDNF* | 1.197923 | 0.001319 |
| *COX7A1* | 1.196717 | 0.005430 |
| *ARHGEF28* | 1.195593 | 0.011563 |
| *FAM155A (NALF1 new name)* | 1.190124 | 0.001865 |
| *IHH* | 1.188725 | 0.002690 |
| *UGT3A2* | 1.186862 | 0.003269 |
| *MYL9* | 1.186368 | 0.002472 |
| *LRP2* | 1.186268 | 0.028674 |
| *EDAR* | 1.184823 | 0.004146 |
| *DPF3* | 1.183963 | 0.004533 |
| *EID3* | 1.183501 | 0.023523 |
| *PRICKLE1* | 1.180543 | 0.004918 |
| *TRIM71* | 1.180531 | 0.005364 |
| *NXN* | 1.177594 | 0.001982 |
| *FIBIN* | 1.177311 | 0.005293 |
| *ATOH8* | 1.175704 | 0.024865 |
| *LIN28B* | 1.175057 | 0.043000 |
| *PPP2R2B* | 1.173115 | 0.007980 |
| *C1S* | 1.171392 | 0.007332 |
| *DTX1* | 1.171246 | 0.026545 |
| *XCL1* | 1.170676 | 0.002575 |
| *SMOC1* | 1.167883 | 0.036270 |
| *COL9A1* | 1.162996 | 0.048066 |
| *GLT1D1* | 1.161552 | 0.002278 |
| *TFAP2A* | 1.158099 | 0.014745 |
| *KCNJ5* | 1.154681 | 0.011994 |
| *PDZD4* | 1.151712 | 0.013978 |
| *OLFML1* | 1.151123 | 0.004268 |
| *ST6GALNAC3* | 1.150670 | 0.026719 |
| *CHRNA3* | 1.150586 | 0.006712 |
| *LIMCH1* | 1.150352 | 0.012102 |
| *LOXL1* | 1.150118 | 0.034656 |
| *CYYR1* | 1.147694 | 0.020485 |
| *ETV5* | 1.147362 | 0.010929 |
| *SGCD* | 1.147263 | 0.013189 |
| *HOXA2* | 1.146642 | 0.013043 |
| *SESN3* | 1.146384 | 0.001912 |
| *GLP1R* | 1.146365 | 0.020122 |
| *TOX2* | 1.145368 | 0.039433 |
| *CLIP3* | 1.143909 | 0.002595 |
| *ECSCR* | 1.143027 | 0.030705 |
| *MYC* | 1.142383 | 0.036299 |
| *MEGF6* | 1.141584 | 0.033582 |
| *ETV4* | 1.140824 | 0.013885 |
| *MRAS* | 1.140214 | 0.004249 |
| *ME1* | 1.138460 | 0.004307 |
| *GLRB* | 1.136878 | 0.009336 |
| *CYSLTR2* | 1.136503 | 0.002012 |
| *PALM2AKAP2* | 1.135205 | 0.005131 |
| *SUCNR1* | 1.132610 | 0.003955 |
| *ID3* | 1.132337 | 0.010157 |
| *DPYSL3* | 1.132094 | 0.006862 |
| *ABCC6* | 1.131450 | 0.048896 |
| *GFRA1* | 1.130533 | 0.002478 |
| *ZEB2* | 1.125615 | 0.022652 |
| *PLXNA4* | 1.122851 | 0.023738 |
| *ISLR2* | 1.121884 | 0.014167 |
| *POGLUT2* | 1.119533 | 0.038331 |
| *MOXD1* | 1.118759 | 0.011910 |
| *RTN4RL1* | 1.117742 | 0.028155 |
| *PARVB* | 1.116722 | 0.014153 |
| *SOX11* | 1.113092 | 0.005153 |
| *SLC5A12* | 1.111262 | 0.003362 |
| *LCTL* | 1.111148 | 0.009203 |
| *SYNPO* | 1.110628 | 0.019308 |
| *CTSL* | 1.106017 | 0.014054 |
| *CPN1* | 1.104835 | 0.016934 |
| *HLA-DRB1* | 1.103876 | 0.033399 |
| *EDEM2* | 1.102468 | 0.018654 |
| *SLC2A8* | 1.101973 | 0.002129 |
| *NR1H4* | 1.100942 | 0.003411 |
| *PCDH7* | 1.100843 | 0.009500 |
| *SMAD9* | 1.100088 | 0.028507 |
| *ATP1B2* | 1.099960 | 0.021689 |
| *CTTNBP2* | 1.099684 | 0.003717 |
| *ADRA2C* | 1.099188 | 0.015726 |
| *SEMA3E* | 1.098580 | 0.025417 |
| *C5orf63* | 1.097868 | 0.017434 |
| *SULT2A1* | 1.095227 | 0.048243 |
| *HACD1* | 1.094155 | 0.002208 |
| *XPNPEP2* | 1.093186 | 0.033049 |
| *SERPINB3* | 1.092813 | 0.012963 |
| *EMILIN2* | 1.091034 | 0.003813 |
| *VCAM1* | 1.090626 | 0.021171 |
| *ANGPT1* | 1.089598 | 0.011522 |
| *B4GAT1* | 1.089196 | 0.002086 |
| *LGI1* | 1.088752 | 0.004579 |
| *ALDH3A2* | 1.088127 | 0.036411 |
| *SHISA9* | 1.086472 | 0.001960 |
| *GPRC5B* | 1.085821 | 0.004023 |
| *GOLIM4* | 1.084406 | 0.041515 |
| *ABCB1* | 1.084224 | 0.002758 |
| *ISL2* | 1.082835 | 0.003601 |
| *POU3F4* | 1.082003 | 0.029126 |
| *SERPINF1* | 1.081361 | 0.010921 |
| *PPM1H* | 1.080813 | 0.011795 |
| *CPVL* | 1.080067 | 0.041940 |
| *ADH6* | 1.078284 | 0.035999 |
| *TNFSF12-TNFSF13* | 1.077806 | 0.001826 |
| *TAC1* | 1.076584 | 0.049640 |
| *NAV3* | 1.073733 | 0.012292 |
| *KIF5C* | 1.073621 | 0.022514 |
| *AGAP2* | 1.070165 | 0.018630 |
| *PRSS23* | 1.069263 | 0.012426 |
| *NAT2* | 1.067648 | 0.015780 |
| *MS4A10* | 1.067634 | 0.002235 |
| *VASN* | 1.063005 | 0.024604 |
| *PDLIM3* | 1.062079 | 0.024365 |
| *CPED1* | 1.061862 | 0.009735 |
| *KLHL4* | 1.057857 | 0.006783 |
| *GYPC* | 1.056041 | 0.004561 |
| *LAMP5* | 1.054734 | 0.007795 |
| *COL17A1* | 1.053660 | 0.013676 |
| *PRRT4* | 1.050463 | 0.004624 |
| *SPARC* | 1.046156 | 0.002819 |
| *LIFR* | 1.044593 | 0.032018 |
| *KHDRBS2* | 1.044511 | 0.040357 |
| *HHEX* | 1.040459 | 0.021090 |
| *EPB41L3* | 1.040217 | 0.004919 |
| *CSDC2* | 1.035182 | 0.006312 |
| *CA3* | 1.033633 | 0.024325 |
| *CCDC152* | 1.033573 | 0.008788 |
| *EMP3* | 1.032848 | 0.024143 |
| *PROCR* | 1.032278 | 0.003321 |
| *ENC1* | 1.030041 | 0.020673 |
| *AGMAT* | 1.029926 | 0.006360 |
| *CA10* | 1.026620 | 0.009652 |
| *C2CD4C* | 1.025759 | 0.033323 |
| *ASGR1* | 1.024341 | 0.009203 |
| *SMIM1* | 1.023396 | 0.025571 |
| *ABCC2* | 1.022091 | 0.021114 |
| *ADAM23* | 1.021584 | 0.003464 |
| *NFE2* | 1.019920 | 0.012958 |
| *PDGFC* | 1.019591 | 0.014452 |
| *WDR86* | 1.019500 | 0.006281 |
| *PREX2* | 1.018390 | 0.010148 |
| *MYO18B* | 1.018386 | 0.003189 |
| *VIT* | 1.017858 | 0.016619 |
| *GRIK1* | 1.017587 | 0.018332 |
| *KCNE4* | 1.013894 | 0.017643 |
| *GPC5* | 1.013371 | 0.013080 |
| *OLFM2* | 1.012845 | 0.002536 |
| *ATP1B1* | 1.010252 | 0.003330 |
| *FGFR1* | 1.007798 | 0.014626 |
| *MYO16* | 1.007168 | 0.009633 |
| *MEGF10* | 1.006160 | 0.014980 |
| *ARPP21* | 1.006118 | 0.015371 |
| *FKBP10* | 1.005879 | 0.003947 |
| *PCDHAC2* | 1.004188 | 0.004193 |
| *PCDH18* | 1.003608 | 0.030795 |
| *KIT* | 1.003465 | 0.044687 |
| *RAB29* | 1.003001 | 0.020488 |
| *TMEM131L* | 1.002776 | 0.004259 |
| *GABRA4* | 1.001421 | 0.002916 |
| *MASP1* | 1.000861 | 0.027190 |
| *PCDH9* | 1.000114 | 0.005019 |
