# Supplementary material for: Identifying miRNA Signatures Associated with Pancreatic Islet Dysfunction in a FOXA2-Deficient iPSC Model
Source: Stem Cell Rev Rep. 2024 Jun 25;20(7):1915–31. doi: 10.1007/s12015-024-10752-0 (PMC11445299; doi:10.1007/s12015-024-10752-0)
Supplement: Supplementary file 4 — Supplementary Material 4 [file 12015_2024_10752_MOESM4_ESM.docx]

**Supplementary Table 4.** Top downregulated DEGs in *FOXA2^–/–^* islets compared with WT-islets (Log2 FC < −1, *P* < 0.05).

| **Gene ID** | **Log2 FC** | ***P*-value** |
| --- | --- | --- |
| *CPB1* | -6.545 | 0.000094 |
| *UPK1A* | -6.440 | 0.000009 |
| *MUC5AC* | -5.636 | 0.000093 |
| *FRZB* | -5.472 | 0.000627 |
| *CXCL8* | -5.110 | 0.000028 |
| *LRRN3* | -4.972 | 0.000111 |
| *INS* | -4.814 | 0.000564 |
| *TMEM265* | -4.780 | 0.003155 |
| *CXCL17* | -4.728 | 0.000015 |
| *PRSS1* | -4.690 | 0.001305 |
| *SCGB1A1* | -4.614 | 0.000443 |
| *PKHD1L1* | -4.600 | 0.000059 |
| *UBD* | -4.498 | 0.000141 |
| *BMP3* | -4.353 | 0.000033 |
| *PPBP* | -4.327 | 0.001568 |
| *RGPD2* | -4.308 | 0.000314 |
| *UPK2* | -4.307 | 0.000533 |
| *ATP2A3* | -4.273 | 0.000063 |
| *PRSS2* | -4.223 | 0.000653 |
| *NKX6.3* | -4.164 | 0.000028 |
| *GCG* | -4.083 | 0.000356 |
| *FER1L6* | -4.067 | 0.020197 |
| *KIAA1324* | -4.040 | 0.000128 |
| *OR2I1P* | -4.011 | 0.000264 |
| *SOX21* | -3.950 | 0.002865 |
| *INSM1* | -3.919 | 0.005020 |
| *MBOAT1* | -3.882 | 0.000020 |
| *COMP* | -3.830 | 0.000453 |
| *PYY* | -3.819 | 0.002394 |
| *MMP10* | -3.812 | 0.028758 |
| *ITM2A* | -3.802 | 0.000076 |
| *HPGD* | -3.786 | 0.003154 |
| *CDH12* | -3.727 | 0.002478 |
| *HS3ST6* | -3.691 | 0.000093 |
| *MUC4* | -3.690 | 0.009579 |
| *LYPD2* | -3.686 | 0.005129 |
| *PAX9* | -3.648 | 0.003233 |
| *ZNF559* | -3.620 | 0.000025 |
| *RPRM* | -3.573 | 0.005719 |
| *MUC1* | -3.572 | 0.000084 |
| *MS4A8* | -3.490 | 0.000069 |
| *MUC20* | -3.490 | 0.000030 |
| *MSMB* | -3.478 | 0.000154 |
| *NTS* | -3.477 | 0.027544 |
| *LRAT* | -3.451 | 0.000176 |
| *CLDN18* | -3.449 | 0.000156 |
| *RGS10* | -3.426 | 0.017489 |
| *PEG3* | -3.422 | 0.000039 |
| *DEGS2* | -3.406 | 0.000031 |
| *EPHA7* | -3.405 | 0.008581 |
| *SLC6A4* | -3.360 | 0.002002 |
| *UCN3* | -3.333 | 0.001773 |
| *CCNO* | -3.329 | 0.032135 |
| *NPW* | -3.316 | 0.004834 |
| *ANXA10* | -3.291 | 0.009944 |
| *TACR1* | -3.282 | 0.003254 |
| *SOX2* | -3.250 | 0.002448 |
| *NEUROG3* | -3.220 | 0.003294 |
| *GMNC* | -3.194 | 0.000033 |
| *AGR3* | -3.173 | 0.000973 |
| *SYK* | -3.167 | 0.000124 |
| *KLK12* | -3.157 | 0.004112 |
| *KLK11* | -3.155 | 0.000450 |
| *RASSF9* | -3.154 | 0.001075 |
| *DEPTOR* | -3.134 | 0.000139 |
| *ZBTB7C* | -3.116 | 0.001055 |
| *GDPD3* | -3.105 | 0.001694 |
| *PCDH19* | -3.100 | 0.001241 |
| *FAM3D* | -3.089 | 0.000950 |
| *ATP13A4* | -3.079 | 0.001806 |
| *LMO3* | -3.078 | 0.002070 |
| *CXCL1* | -3.048 | 0.001018 |
| *ACSL1* | -3.046 | 0.001702 |
| *MMP7* | -3.008 | 0.007840 |
| *GRHL3* | -3.006 | 0.000965 |
| *S100P* | -2.987 | 0.001245 |
| *ZNF208* | -2.981 | 0.004176 |
| *ZNF471* | -2.975 | 0.000890 |
| *ZNF667* | -2.966 | 0.000051 |
| *KLK10* | -2.965 | 0.000084 |
| *MSLN* | -2.964 | 0.000478 |
| *SGPP2* | -2.958 | 0.000072 |
| *PERCC1* | -2.941 | 0.000131 |
| *GJB7* | -2.940 | 0.000225 |
| *IGFBP1* | -2.926 | 0.012484 |
| *RARRES2* | -2.924 | 0.014587 |
| *DLL1* | -2.908 | 0.000551 |
| *NKX2-2* | -2.897 | 0.008452 |
| *GABRA1* | -2.890 | 0.011704 |
| *UGT2B15* | -2.888 | 0.045793 |
| *PAPLN* | -2.883 | 0.003091 |
| *IL1A* | -2.878 | 0.010967 |
| *MYBPC1* | -2.877 | 0.003957 |
| *UPK3B* | -2.865 | 0.018825 |
| *EHF* | -2.841 | 0.000909 |
| *ZACN* | -2.830 | 0.027083 |
| *CNTN3* | -2.825 | 0.021109 |
| *TMSB15A* | -2.819 | 0.016865 |
| *AFF2* | -2.791 | 0.000194 |
| *FUT3* | -2.789 | 0.000167 |
| *C10orf99* | -2.782 | 0.001155 |
| *KRT7* | -2.780 | 0.002027 |
| *NTN1* | -2.767 | 0.000277 |
| *KLK1* | -2.767 | 0.000763 |
| *CXCL3* | -2.763 | 0.000079 |
| *HEPACAM2* | -2.755 | 0.000262 |
| *LCK* | -2.734 | 0.000246 |
| *MLPH* | -2.701 | 0.003326 |
| *RND1* | -2.695 | 0.000588 |
| *FA2H* | -2.678 | 0.000104 |
| *ZNF676* | -2.657 | 0.000369 |
| *ASCL1* | -2.654 | 0.025424 |
| *SAMD13* | -2.647 | 0.000092 |
| *PDE8B* | -2.646 | 0.000349 |
| *NXPH2* | -2.644 | 0.000310 |
| *RGPD1* | -2.638 | 0.000907 |
| *FAM149A* | -2.638 | 0.000065 |
| *SYBU* | -2.637 | 0.001298 |
| *ITGA4* | -2.618 | 0.027904 |
| *ATP6V1B1* | -2.598 | 0.000273 |
| *IL23A* | -2.592 | 0.006872 |
| *CRIP1* | -2.573 | 0.005462 |
| *MCIDAS* | -2.570 | 0.044623 |
| *KCNK16* | -2.564 | 0.000834 |
| *TMPRSS4* | -2.558 | 0.003784 |
| *GGT6* | -2.550 | 0.004181 |
| *NIBAN1* | -2.549 | 0.000257 |
| *GNA15* | -2.539 | 0.006887 |
| *PLCE1* | -2.535 | 0.001097 |
| *ZNF736* | -2.534 | 0.000090 |
| *UNC13C* | -2.530 | 0.001950 |
| *PCDHA6* | -2.515 | 0.000315 |
| *GFRA3* | -2.497 | 0.005359 |
| *PTF1A* | -2.496 | 0.022354 |
| *TNFRSF11B* | -2.494 | 0.003990 |
| *PTPRT* | -2.480 | 0.004472 |
| *RNF223* | -2.474 | 0.000085 |
| *SLC30A2* | -2.472 | 0.000162 |
| *KRTAP2-3* | -2.463 | 0.020646 |
| *AR* | -2.454 | 0.000132 |
| *SUSD4* | -2.451 | 0.001329 |
| *ALDH1A3* | -2.450 | 0.000733 |
| *CAMK2B* | -2.441 | 0.000227 |
| *ATP10B* | -2.437 | 0.005857 |
| *BIK* | -2.429 | 0.008291 |
| *GUCA2B* | -2.424 | 0.003863 |
| *COL22A1* | -2.423 | 0.003374 |
| *ZNF726* | -2.414 | 0.000436 |
| *APOL4* | -2.397 | 0.000208 |
| *CELSR1* | -2.396 | 0.000290 |
| *ADGRF1* | -2.376 | 0.000223 |
| *VSIG1* | -2.366 | 0.042825 |
| *FAM83E* | -2.343 | 0.000128 |
| *TRIB3* | -2.340 | 0.001052 |
| *ZNF177* | -2.338 | 0.006792 |
| *IL20RA* | -2.321 | 0.000292 |
| *SMIM5* | -2.317 | 0.001982 |
| *UPK3BL1* | -2.316 | 0.003461 |
| *TRIM36* | -2.311 | 0.008072 |
| *MYB* | -2.299 | 0.010419 |
| *ALDH3B2* | -2.299 | 0.012061 |
| *SDCBP2* | -2.296 | 0.008590 |
| *NHS* | -2.294 | 0.034727 |
| *UGT2B17* | -2.287 | 0.021883 |
| *RNASE4* | -2.286 | 0.000478 |
| *PALMD* | -2.282 | 0.044211 |
| *PNPO* | -2.281 | 0.001244 |
| *SPTSSB* | -2.276 | 0.010386 |
| *C16orf89* | -2.273 | 0.000258 |
| *ABO* | -2.269 | 0.011387 |
| *TMPRSS13* | -2.268 | 0.001549 |
| *LPAR3* | -2.243 | 0.012714 |
| *LINGO2* | -2.237 | 0.000351 |
| *LURAP1L* | -2.228 | 0.001379 |
| *CXCL5* | -2.226 | 0.001216 |
| *CAMK1D* | -2.225 | 0.007096 |
| *ANG* | -2.223 | 0.000668 |
| *HES6* | -2.223 | 0.022610 |
| *VSIG2* | -2.222 | 0.005441 |
| *OTX1* | -2.221 | 0.000320 |
| *SMIM22* | -2.220 | 0.000620 |
| *LHX5* | -2.216 | 0.000359 |
| *LPAR5* | -2.206 | 0.000290 |
| *NECTIN4* | -2.203 | 0.001683 |
| *GAST* | -2.192 | 0.026778 |
| *P2RX1* | -2.179 | 0.001702 |
| *RNF224* | -2.178 | 0.001098 |
| *LAMB3* | -2.176 | 0.026730 |
| *RASSF2* | -2.163 | 0.004210 |
| *LMX1B* | -2.154 | 0.013177 |
| *KRT6B* | -2.153 | 0.015350 |
| *CHRM1* | -2.141 | 0.001047 |
| *SHH* | -2.138 | 0.000222 |
| *FOLH1* | -2.130 | 0.014639 |
| *PPY* | -2.130 | 0.006051 |
| *SEMA4A* | -2.121 | 0.001269 |
| *CLDN10* | -2.111 | 0.034158 |
| *BCAS1* | -2.104 | 0.001418 |
| *RIMKLA* | -2.102 | 0.001543 |
| *EVPL* | -2.100 | 0.000685 |
| *RNF186* | -2.099 | 0.001328 |
| *SMPD3* | -2.097 | 0.000398 |
| *PCSK2* | -2.093 | 0.001923 |
| *SERPINB2* | -2.090 | 0.047196 |
| *KIF19* | -2.085 | 0.015270 |
| *MYO5B* | -2.082 | 0.000829 |
| *CPM* | -2.079 | 0.018724 |
| *S100A2* | -2.075 | 0.000153 |
| *SPACA4* | -2.073 | 0.027058 |
| *TMEM100* | -2.071 | 0.017443 |
| *LTB* | -2.068 | 0.006267 |
| *SLC38A3* | -2.068 | 0.000314 |
| *IRF5* | -2.066 | 0.000324 |
| *IL12A* | -2.061 | 0.005140 |
| *DPY19L2* | -2.051 | 0.006344 |
| *ASTL* | -2.049 | 0.000373 |
| *SEMA3A* | -2.042 | 0.001207 |
| *EFEMP1* | -2.042 | 0.001534 |
| *RFX6* | -2.042 | 0.001358 |
| *TFF1* | -2.040 | 0.029253 |
| *ZNF835* | -2.037 | 0.000277 |
| *BAIAP3* | -2.033 | 0.008062 |
| *AOC1* | -2.030 | 0.003640 |
| *FAM167A* | -2.029 | 0.015739 |
| *C2orf72* | -2.028 | 0.000444 |
| *TAAR1* | -2.027 | 0.002781 |
| *PLCL2* | -2.024 | 0.000212 |
| *UCP2* | -2.013 | 0.006024 |
| *RIPPLY3* | -2.011 | 0.004640 |
| *SEMA3F* | -2.008 | 0.001696 |
| *NKX6.1* | -2.008 | 0.020767 |
| *NOL4* | -2.007 | 0.000217 |
| *SLC25A12* | -2.001 | 0.001692 |
| *MLXIPL* | -1.997 | 0.001945 |
| *GNA14* | -1.996 | 0.024093 |
| *SPINK1* | -1.995 | 0.002861 |
| *KLF5* | -1.994 | 0.000617 |
| *CACNG4* | -1.994 | 0.000419 |
| *LRRC61* | -1.977 | 0.001890 |
| *OVOL2* | -1.976 | 0.035332 |
| *TMCC2* | -1.962 | 0.020002 |
| *CNTNAP3* | -1.961 | 0.000230 |
| *PTGS2* | -1.960 | 0.003701 |
| *LRRC10B* | -1.959 | 0.041431 |
| *ZXDA* | -1.957 | 0.000187 |
| *VWA5B2* | -1.952 | 0.001372 |
| *THRB* | -1.950 | 0.009821 |
| *GDF15* | -1.947 | 0.000501 |
| *FUT1* | -1.945 | 0.000782 |
| *SEMA3C* | -1.938 | 0.011795 |
| *CYP4F12* | -1.938 | 0.022220 |
| *CLIC5* | -1.935 | 0.017618 |
| *PLA2G4B* | -1.932 | 0.015796 |
| *SLC34A3* | -1.930 | 0.001550 |
| *ARHGAP30* | -1.929 | 0.000384 |
| *SPIB* | -1.928 | 0.003433 |
| *MYLPF* | -1.927 | 0.047784 |
| *C6orf141* | -1.921 | 0.014020 |
| *PID1* | -1.920 | 0.002186 |
| *GPBAR1* | -1.914 | 0.000765 |
| *EMID1* | -1.914 | 0.010140 |
| *TNF* | -1.913 | 0.002522 |
| *LRRC4* | -1.913 | 0.000229 |
| *ACSS1* | -1.908 | 0.000465 |
| *HIF3A* | -1.902 | 0.004680 |
| *HSPE1-MOB4* | -1.902 | 0.030515 |
| *PLAUR* | -1.899 | 0.003615 |
| *CDH9* | -1.896 | 0.014012 |
| *TRPM4* | -1.895 | 0.000285 |
| *CDH7* | -1.895 | 0.025479 |
| *KCNN1* | -1.892 | 0.006227 |
| *LCN2* | -1.892 | 0.000541 |
| *DLL4* | -1.891 | 0.001372 |
| *FRY* | -1.890 | 0.002506 |
| *COL14A1* | -1.889 | 0.019458 |
| *FERMT1* | -1.885 | 0.027903 |
| *PCDHGA10* | -1.881 | 0.002355 |
| *GABRB2* | -1.881 | 0.023961 |
| *PLCH2* | -1.880 | 0.000802 |
| *DMBT1* | -1.877 | 0.000429 |
| *PANX2* | -1.874 | 0.002072 |
| *TPBG* | -1.869 | 0.000695 |
| *MUC5B* | -1.867 | 0.017076 |
| *CCL20* | -1.861 | 0.020581 |
| *SNTB1* | -1.852 | 0.001054 |
| *TMEM156* | -1.850 | 0.005598 |
| *PRODH* | -1.847 | 0.000411 |
| *MISP* | -1.844 | 0.001439 |
| *ALOX15* | -1.838 | 0.028500 |
| *KIAA0319* | -1.835 | 0.005467 |
| *SPRR2A* | -1.834 | 0.001655 |
| *BMPR1B* | -1.833 | 0.000304 |
| *EBF4* | -1.833 | 0.002013 |
| *KLF4* | -1.826 | 0.007130 |
| *IRAK2* | -1.822 | 0.023421 |
| *UPP1* | -1.821 | 0.040542 |
| *ATG16L2* | -1.820 | 0.013471 |
| *IQANK1* | -1.819 | 0.004081 |
| *UPK3BL2* | -1.819 | 0.000265 |
| *CDHR1* | -1.818 | 0.002940 |
| *PHACTR3* | -1.818 | 0.010215 |
| *PCDH1* | -1.813 | 0.027435 |
| *EXPH5* | -1.812 | 0.001166 |
| *COL6A2* | -1.807 | 0.006240 |
| *CAPS* | -1.803 | 0.000588 |
| *ABCG1* | -1.797 | 0.001020 |
| *NR3C2* | -1.796 | 0.005904 |
| *SIDT1* | -1.795 | 0.000771 |
| *ARAP3* | -1.794 | 0.018435 |
| *SCNN1A* | -1.794 | 0.028341 |
| *DACH2* | -1.792 | 0.001283 |
| *DSTN* | -1.790 | 0.019380 |
| *FOXQ1* | -1.790 | 0.021329 |
| *NEUROD1* | -1.789 | 0.006574 |
| *CXCL2* | -1.786 | 0.005516 |
| *SEMA7A* | -1.783 | 0.030450 |
| *MT1G* | -1.780 | 0.049183 |
| *NPY1R* | -1.775 | 0.033571 |
| *JUNB* | -1.772 | 0.001907 |
| *RELB* | -1.770 | 0.003850 |
| *KNDC1* | -1.768 | 0.001944 |
| *TNFRSF14* | -1.759 | 0.003019 |
| *RAPGEFL1* | -1.758 | 0.000874 |
| *THBD* | -1.757 | 0.000390 |
| *IL32* | -1.756 | 0.007821 |
| *NRXN1* | -1.755 | 0.001037 |
| *DOCK3* | -1.750 | 0.010172 |
| *VGLL1* | -1.750 | 0.018871 |
| *ADGRG2* | -1.749 | 0.019929 |
| *LTBP4* | -1.749 | 0.011985 |
| *ICAM4* | -1.748 | 0.046341 |
| *DNAJC12* | -1.748 | 0.006754 |
| *PRKCG* | -1.740 | 0.013747 |
| *APOH* | -1.739 | 0.036507 |
| *NXPH4* | -1.737 | 0.022630 |
| *PAQR6* | -1.735 | 0.010609 |
| *GRB14* | -1.731 | 0.004872 |
| *UGDH* | -1.729 | 0.001938 |
| *PLAAT4* | -1.728 | 0.007241 |
| *GALNT18* | -1.728 | 0.008630 |
| *TGM1* | -1.726 | 0.001106 |
| *CRACR2B* | -1.721 | 0.000633 |
| *SCPEP1* | -1.721 | 0.015748 |
| *GPM6B* | -1.721 | 0.006094 |
| *TCTEX1D4* | -1.718 | 0.000696 |
| *SLFN13* | -1.718 | 0.007944 |
| *FAM174B* | -1.716 | 0.000811 |
| *POMC* | -1.715 | 0.019984 |
| *SYNE2* | -1.714 | 0.002197 |
| *TNNT1* | -1.711 | 0.018566 |
| *KIF21B* | -1.708 | 0.021579 |
| *KLHL35* | -1.704 | 0.001337 |
| *USP6* | -1.702 | 0.005130 |
| *F3* | -1.700 | 0.030851 |
| *C19orf33* | -1.699 | 0.001135 |
| *WNT4* | -1.690 | 0.024940 |
| *RAMP1* | -1.689 | 0.005853 |
| *SMAGP* | -1.688 | 0.006273 |
| *MAP3K5* | -1.688 | 0.002158 |
| *ITGA2B* | -1.687 | 0.013580 |
| *TNFSF9* | -1.686 | 0.001146 |
| *ZBED6CL* | -1.681 | 0.002556 |
| *SLC22A9* | -1.675 | 0.004789 |
| *CGB8* | -1.673 | 0.012099 |
| *TSPAN12* | -1.671 | 0.015625 |
| *C9orf152* | -1.671 | 0.002636 |
| *GALR2* | -1.670 | 0.043973 |
| *SLC16A11* | -1.668 | 0.002473 |
| *PDLIM4* | -1.666 | 0.038097 |
| *METTL7A* | -1.665 | 0.003765 |
| *SLC30A8* | -1.663 | 0.002418 |
| *ACP7* | -1.660 | 0.000585 |
| *KLHL1* | -1.652 | 0.002767 |
| *STYK1* | -1.651 | 0.001026 |
| *SGSM1* | -1.650 | 0.000517 |
| *ZNF257* | -1.650 | 0.000362 |
| *PCDHB5* | -1.647 | 0.002952 |
| *TAFA3* | -1.643 | 0.023058 |
| *EPB41L4A* | -1.642 | 0.013227 |
| *PLEK2* | -1.641 | 0.016646 |
| *ADAM8* | -1.639 | 0.004164 |
| *PLAU* | -1.637 | 0.008937 |
| *GPSM3* | -1.637 | 0.002378 |
| *ZNF229* | -1.637 | 0.000381 |
| *TMEM79* | -1.636 | 0.000400 |
| *ATF3* | -1.635 | 0.000555 |
| *CELF3* | -1.633 | 0.005517 |
| *TACC2* | -1.629 | 0.002510 |
| *SLC15A2* | -1.624 | 0.013754 |
| *DOCK5* | -1.615 | 0.031724 |
| *ARX* | -1.613 | 0.045159 |
| *SLFN5* | -1.612 | 0.004258 |
| *ZC3H12A* | -1.609 | 0.000533 |
| *TEN1-CDK3* | -1.607 | 0.003615 |
| *PER2* | -1.600 | 0.001656 |
| *AOX1* | -1.595 | 0.019656 |
| *EPN3* | -1.595 | 0.000573 |
| *ZNF69* | -1.590 | 0.003002 |
| *TGFA* | -1.590 | 0.000986 |
| *LFNG* | -1.589 | 0.002395 |
| *ZBTB42* | -1.588 | 0.001452 |
| *EPHA10* | -1.587 | 0.004934 |
| *TREX1* | -1.584 | 0.017851 |
| *ZG16B* | -1.577 | 0.000451 |
| *TMEM176A* | -1.575 | 0.003318 |
| *TRIM47* | -1.571 | 0.006807 |
| *ENTPD2* | -1.571 | 0.006145 |
| *SLC9A2* | -1.570 | 0.004095 |
| *ADH1A* | -1.569 | 0.003276 |
| *REEP1* | -1.568 | 0.004048 |
| *TNNC1* | -1.567 | 0.033675 |
| *IGFL1* | -1.563 | 0.001179 |
| *TRIM38* | -1.561 | 0.002410 |
| *TPH1* | -1.560 | 0.001016 |
| *C20orf204* | -1.560 | 0.030023 |
| *ARRDC3* | -1.556 | 0.002320 |
| *KRT81* | -1.556 | 0.001395 |
| *RAB3D* | -1.553 | 0.001045 |
| *IKZF2* | -1.551 | 0.002351 |
| *FXYD4* | -1.550 | 0.001318 |
| *IFI44* | -1.550 | 0.007901 |
| *HPSE* | -1.550 | 0.001533 |
| *OAS3* | -1.549 | 0.019931 |
| *FAM160A1* | -1.548 | 0.001030 |
| *SYT5* | -1.546 | 0.016911 |
| *ERO1B* | -1.544 | 0.003526 |
| *CAPN5* | -1.544 | 0.001028 |
| *GJA1* | -1.540 | 0.001632 |
| *ACTL10* | -1.539 | 0.009968 |
| *TFF3* | -1.538 | 0.001947 |
| *TNFRSF11A* | -1.538 | 0.003172 |
| *STX19* | -1.535 | 0.000482 |
| *OXGR1* | -1.534 | 0.025014 |
| *GALNT12* | -1.532 | 0.001709 |
| *POF1B* | -1.531 | 0.022017 |
| *FBP1* | -1.531 | 0.008191 |
| *CYP2C18* | -1.529 | 0.018006 |
| *OLFM4* | -1.526 | 0.017430 |
| *B4GALNT3* | -1.526 | 0.008377 |
| *TMCO4* | -1.524 | 0.003658 |
| *CDKN1B* | -1.524 | 0.014230 |
| *KLK13* | -1.521 | 0.003769 |
| *ATP13A5* | -1.519 | 0.002778 |
| *GCNT1* | -1.519 | 0.015917 |
| *RASSF6* | -1.517 | 0.033911 |
| *ZFP36L1* | -1.515 | 0.001275 |
| *TRIM45* | -1.514 | 0.003811 |
| *WNK2* | -1.514 | 0.000870 |
| *E2F7* | -1.514 | 0.002304 |
| *GRHL1* | -1.510 | 0.000923 |
| *GALNT14* | -1.506 | 0.002348 |
| *ADGRG5* | -1.503 | 0.004548 |
| *AMOT* | -1.503 | 0.008357 |
| *ANGPT2* | -1.501 | 0.009879 |
| *UBA7* | -1.500 | 0.009183 |
| *AREG* | -1.500 | 0.012832 |
| *RUNX2* | -1.499 | 0.010266 |
| *SCG3* | -1.498 | 0.028335 |
| *MFNG* | -1.496 | 0.003282 |
| *HSH2D* | -1.493 | 0.004141 |
| *RNF183* | -1.488 | 0.023980 |
| *C1orf127* | -1.486 | 0.000841 |
| *NEXMIF* | -1.485 | 0.035935 |
| *C20orf194* | -1.483 | 0.002156 |
| *TBC1D8* | -1.483 | 0.000997 |
| *CHD3* | -1.478 | 0.001971 |
| *TNFAIP3* | -1.477 | 0.004433 |
| *ZFP36* | -1.476 | 0.002616 |
| *TMEM255A* | -1.476 | 0.038011 |
| *SLC10A5* | -1.474 | 0.005407 |
| *C15orf39* | -1.474 | 0.000970 |
| *GIPR* | -1.471 | 0.046349 |
| *RARB* | -1.471 | 0.024582 |
| *KCNH6* | -1.471 | 0.012661 |
| *OXTR* | -1.470 | 0.005707 |
| *SPDEF* | -1.469 | 0.003887 |
| *S100A6* | -1.468 | 0.001581 |
| *HS6ST2* | -1.463 | 0.004457 |
| *CDO1* | -1.462 | 0.003938 |
| *VNN2* | -1.462 | 0.013233 |
| *FOXN1* | -1.461 | 0.003661 |
| *SLITRK6* | -1.461 | 0.009643 |
| *USP41* | -1.460 | 0.025163 |
| *PTPRN2* | -1.458 | 0.044138 |
| *EGLN3* | -1.456 | 0.021643 |
| *CCN3* | -1.455 | 0.014954 |
| *ADCY7* | -1.455 | 0.035309 |
| *PAX4* | -1.455 | 0.046518 |
| *FSTL5* | -1.453 | 0.015670 |
| *SULT1A3* | -1.453 | 0.007054 |
| *GAB2* | -1.452 | 0.004117 |
| *LBH* | -1.451 | 0.003241 |
| *TRIM16* | -1.449 | 0.001965 |
| *ADCY1* | -1.446 | 0.002243 |
| *IRF1* | -1.442 | 0.001858 |
| *AMIGO2* | -1.441 | 0.001760 |
| *JPH1* | -1.439 | 0.036780 |
| *ZNF572* | -1.439 | 0.002002 |
| *MYOM3* | -1.438 | 0.015313 |
| *CDKN1A* | -1.438 | 0.002046 |
| *TMEM176B* | -1.437 | 0.007239 |
| *PRSS16* | -1.437 | 0.009003 |
| *ABCA3* | -1.437 | 0.004396 |
| *ZNF528* | -1.437 | 0.035965 |
| *CDCP1* | -1.435 | 0.043382 |
| *ZNF860* | -1.432 | 0.017763 |
| *VWA2* | -1.432 | 0.004627 |
| *MTMR7* | -1.431 | 0.000798 |
| *C3orf14* | -1.431 | 0.046570 |
| *GBP6* | -1.430 | 0.034570 |
| *STAC* | -1.426 | 0.002697 |
| *CKMT1A* | -1.425 | 0.001904 |
| *KCNS3* | -1.424 | 0.001009 |
| *S100A4* | -1.419 | 0.018572 |
| *FAM167B* | -1.418 | 0.023868 |
| *SLC7A2* | -1.418 | 0.002289 |
| *B3GALT4* | -1.418 | 0.000975 |
| *PRR15L* | -1.417 | 0.004639 |
| *SCGN* | -1.417 | 0.002077 |
| *SFMBT1* | -1.415 | 0.001304 |
| *LGR4* | -1.414 | 0.002253 |
| *SLC52A3* | -1.413 | 0.003278 |
| *SUSD6* | -1.413 | 0.010001 |
| *MYH14* | -1.412 | 0.002230 |
| *IQSEC2* | -1.409 | 0.024659 |
| *IFIT3* | -1.404 | 0.005309 |
| *ANKRD22* | -1.401 | 0.007175 |
| *CRYBG1* | -1.400 | 0.002074 |
| *FGD6* | -1.399 | 0.044040 |
| *SPIRE2* | -1.398 | 0.029198 |
| *PDX1* | -1.392 | 0.004558 |
| *SPPL2B* | -1.391 | 0.003989 |
| *TTC9* | -1.389 | 0.006876 |
| *RIBC2* | -1.389 | 0.017886 |
| *PAQR4* | -1.388 | 0.040263 |
| *GJB3* | -1.388 | 0.012975 |
| *SIT1* | -1.387 | 0.023822 |
| *BFSP1* | -1.382 | 0.041800 |
| *NBL1* | -1.382 | 0.010305 |
| *CADM2* | -1.382 | 0.001277 |
| *AHNAK2* | -1.380 | 0.045924 |
| *GCGR* | -1.380 | 0.013826 |
| *BCL2L14* | -1.378 | 0.006057 |
| *SLAMF7* | -1.377 | 0.019499 |
| *CD9* | -1.376 | 0.005930 |
| *MCTS2P* | -1.374 | 0.009316 |
| *IKBKE* | -1.372 | 0.011074 |
| *APOL1* | -1.372 | 0.044488 |
| *C21orf58* | -1.371 | 0.000787 |
| *MEOX1* | -1.367 | 0.000992 |
| *CHGA* | -1.365 | 0.026737 |
| *GCOM1* | -1.364 | 0.016783 |
| *SLC37A1* | -1.363 | 0.010094 |
| *ARRDC2* | -1.360 | 0.001894 |
| *HAUS4* | -1.360 | 0.005784 |
| *RUNX1* | -1.357 | 0.001564 |
| *TFCP2L1* | -1.356 | 0.003096 |
| *KCNH8* | -1.355 | 0.023042 |
| *ESRP2* | -1.353 | 0.002671 |
| *TACSTD2* | -1.350 | 0.003080 |
| *MYEOV* | -1.350 | 0.040870 |
| *TXNIP* | -1.350 | 0.015325 |
| *LRP11* | -1.350 | 0.029113 |
| *DOCK11* | -1.349 | 0.001677 |
| *NR4A2* | -1.345 | 0.006674 |
| *VPS37B* | -1.345 | 0.017607 |
| *ACPP* | -1.345 | 0.023512 |
| *ABCC8* | -1.342 | 0.004895 |
| *STAP2* | -1.341 | 0.000924 |
| *MAST4* | -1.340 | 0.019528 |
| *KCNK13* | -1.339 | 0.033592 |
| *CCDC187* | -1.338 | 0.023572 |
| *GPCPD1* | -1.337 | 0.002040 |
| *ZDHHC14* | -1.335 | 0.031918 |
| *KISS1* | -1.335 | 0.003537 |
| *MYZAP* | -1.334 | 0.012427 |
| *NR2F1* | -1.334 | 0.017207 |
| *MYO10* | -1.334 | 0.001229 |
| *AQP3* | -1.333 | 0.003207 |
| *ST8SIA2* | -1.333 | 0.007189 |
| *LTK* | -1.332 | 0.018947 |
| *FAM43A* | -1.328 | 0.016143 |
| *N4BP3* | -1.328 | 0.001296 |
| *NLRP2* | -1.328 | 0.001010 |
| *XK* | -1.326 | 0.001311 |
| *CKMT1B* | -1.325 | 0.009791 |
| *CHST15* | -1.325 | 0.005334 |
| *SGSM2* | -1.324 | 0.000864 |
| *IER5* | -1.322 | 0.014909 |
| *PTPN13* | -1.320 | 0.002214 |
| *BLNK* | -1.319 | 0.008030 |
| *CPT1A* | -1.317 | 0.008920 |
| *FFAR1* | -1.316 | 0.000939 |
| *MGME1* | -1.312 | 0.001774 |
| *SLC16A5* | -1.311 | 0.004397 |
| *CLDN4* | -1.310 | 0.001050 |
| *SLC4A11* | -1.307 | 0.033926 |
| *BSPRY* | -1.307 | 0.001753 |
| *ADCY2* | -1.306 | 0.007542 |
| *CLCNKB* | -1.303 | 0.014630 |
| *ARNTL2* | -1.303 | 0.001964 |
| *TMTC2* | -1.302 | 0.014278 |
| *TRIM22* | -1.302 | 0.003237 |
| *PCDHB8* | -1.301 | 0.008582 |
| *KISS1R* | -1.300 | 0.024383 |
| *ENO2* | -1.299 | 0.031085 |
| *ALG1L* | -1.298 | 0.042150 |
| *CBLC* | -1.296 | 0.003577 |
| *CASZ1* | -1.296 | 0.007891 |
| *SYT7* | -1.296 | 0.006145 |
| *HID1* | -1.292 | 0.004379 |
| *LYPD6B* | -1.292 | 0.004131 |
| *CPLX1* | -1.291 | 0.002596 |
| *C15orf62* | -1.291 | 0.003111 |
| *C1orf116* | -1.290 | 0.014497 |
| *CCDC154* | -1.289 | 0.001366 |
| *PSCA* | -1.288 | 0.003341 |
| *AQP5* | -1.286 | 0.046170 |
| *SLCO3A1* | -1.286 | 0.020220 |
| *RECQL5* | -1.285 | 0.024966 |
| *MYCL* | -1.285 | 0.025711 |
| *HES1* | -1.284 | 0.037253 |
| *TLR5* | -1.284 | 0.036120 |
| *PHYHD1* | -1.283 | 0.007589 |
| *IL17RE* | -1.281 | 0.001809 |
| *SPRR2D* | -1.280 | 0.003418 |
| *PLSCR1* | -1.278 | 0.038482 |
| *CYP2D6* | -1.278 | 0.028252 |
| *TASP1* | -1.277 | 0.004311 |
| *CHGB* | -1.277 | 0.024924 |
| *NUDT19* | -1.277 | 0.038553 |
| *ANGPTL4* | -1.277 | 0.026593 |
| *ZFP28* | -1.276 | 0.001575 |
| *MTUS2* | -1.275 | 0.001711 |
| *MEIS3* | -1.275 | 0.037301 |
| *TM4SF1* | -1.274 | 0.001406 |
| *QSOX1* | -1.274 | 0.029289 |
| *PITX1* | -1.272 | 0.001694 |
| *ENTPD8* | -1.272 | 0.044908 |
| *A4GALT* | -1.271 | 0.006219 |
| *PLAAT5* | -1.269 | 0.019926 |
| *PTAFR* | -1.268 | 0.031502 |
| *RBCK1* | -1.266 | 0.003171 |
| *MPP7* | -1.266 | 0.009161 |
| *CACNA1A* | -1.261 | 0.004077 |
| *JMJD7-PLA2G4B* | -1.261 | 0.005453 |
| *EPHX3* | -1.261 | 0.012838 |
| *MCF2L* | -1.260 | 0.005826 |
| *DUSP10* | -1.258 | 0.005786 |
| *TMEM121B* | -1.257 | 0.005964 |
| *TM6SF1* | -1.257 | 0.047085 |
| *MSH5* | -1.256 | 0.011934 |
| *CBFA2T3* | -1.255 | 0.006803 |
| *OVOL1* | -1.255 | 0.009826 |
| *NOTCH2NLC* | -1.255 | 0.001353 |
| *GPHA2* | -1.255 | 0.038673 |
| *CDHR3* | -1.254 | 0.004311 |
| *FAM102B* | -1.251 | 0.011376 |
| *ARSJ* | -1.249 | 0.021586 |
| *MOCOS* | -1.247 | 0.001293 |
| *CBLN3* | -1.247 | 0.003203 |
| *NOTCH2NLB* | -1.246 | 0.019310 |
| *TAP1* | -1.243 | 0.022793 |
| *OTULINL* | -1.240 | 0.002993 |
| *TFAP2E* | -1.240 | 0.003936 |
| *PAIP2B* | -1.240 | 0.005252 |
| *ELF3* | -1.240 | 0.001102 |
| *FAM110A* | -1.240 | 0.001217 |
| *LMO7* | -1.239 | 0.008362 |
| *SLC41A2* | -1.238 | 0.023930 |
| *PCED1A* | -1.238 | 0.007308 |
| *ZNF559-ZNF177* | -1.236 | 0.001133 |
| *IGSF11* | -1.236 | 0.004428 |
| *DGKD* | -1.235 | 0.003855 |
| *TCF19* | -1.235 | 0.012547 |
| *NR3C1* | -1.233 | 0.001518 |
| *PLEKHG4B* | -1.233 | 0.001188 |
| *TPD52* | -1.231 | 0.003784 |
| *ITPKA* | -1.231 | 0.011748 |
| *CRYBA2* | -1.231 | 0.019914 |
| *BHLHE40* | -1.228 | 0.047768 |
| *TK1* | -1.228 | 0.045019 |
| *ARHGAP32* | -1.228 | 0.008827 |
| *PPP1R13L* | -1.228 | 0.010462 |
| *TRIM14* | -1.225 | 0.014026 |
| *TMEM163* | -1.223 | 0.003059 |
| *EBAG9* | -1.223 | 0.005029 |
| *PSD4* | -1.222 | 0.006607 |
| *LYPD6* | -1.221 | 0.005875 |
| *IFITM1* | -1.220 | 0.015091 |
| *MACC1* | -1.218 | 0.018311 |
| *SALL4* | -1.217 | 0.003436 |
| *ZNF844* | -1.217 | 0.001193 |
| *SH3RF1* | -1.217 | 0.002883 |
| *ARRB1* | -1.216 | 0.001201 |
| *GSDMD* | -1.214 | 0.005216 |
| *TMEM54* | -1.214 | 0.001855 |
| *USP54* | -1.214 | 0.010158 |
| *LRRC26* | -1.214 | 0.042423 |
| *PLA2G4F* | -1.213 | 0.003122 |
| *LTBP2* | -1.212 | 0.006728 |
| *ACE* | -1.212 | 0.009953 |
| *MISP3* | -1.211 | 0.003912 |
| *ETV7* | -1.211 | 0.024882 |
| *DOCK6* | -1.210 | 0.007924 |
| *TRIM25* | -1.210 | 0.004433 |
| *ISG20* | -1.209 | 0.038940 |
| *SMIM14* | -1.209 | 0.007232 |
| *PSMB8* | -1.206 | 0.004600 |
| *CCDC188* | -1.205 | 0.010635 |
| *SCN3A* | -1.203 | 0.001632 |
| *IQGAP3* | -1.202 | 0.016648 |
| *H2BC21* | -1.202 | 0.006356 |
| *DENND11* | -1.201 | 0.013209 |
| *CHRNB1* | -1.200 | 0.001406 |
| *ZNF714* | -1.199 | 0.001774 |
| *DEPP1* | -1.195 | 0.011827 |
| *FAM83B* | -1.192 | 0.001950 |
| *VANGL1* | -1.191 | 0.009471 |
| *CDKN2C* | -1.191 | 0.041811 |
| *ARHGAP6* | -1.189 | 0.013606 |
| *PLEKHM3* | -1.188 | 0.006671 |
| *BRINP2* | -1.187 | 0.004569 |
| *P2RY1* | -1.186 | 0.002905 |
| *DEFB4A* | -1.185 | 0.030601 |
| *SP100* | -1.185 | 0.003582 |
| *MSH5-SAPCD1* | -1.183 | 0.003686 |
| *CACNG6* | -1.183 | 0.007517 |
| *IRF6* | -1.183 | 0.002395 |
| *TRIM34* | -1.182 | 0.027458 |
| *SQOR* | -1.181 | 0.015999 |
| *TRIM4* | -1.181 | 0.004677 |
| *MAGI2* | -1.180 | 0.002286 |
| *MAFF* | -1.179 | 0.020888 |
| *ANO8* | -1.178 | 0.015659 |
| *ZBED2* | -1.176 | 0.001721 |
| *NFKB2* | -1.176 | 0.021729 |
| *FOSL2* | -1.176 | 0.003779 |
| *H2BU1* | -1.172 | 0.040346 |
| *CCDC6* | -1.168 | 0.011111 |
| *FAM50B* | -1.167 | 0.001700 |
| *STN1* | -1.167 | 0.001454 |
| *SMOX* | -1.167 | 0.010008 |
| *RGS2* | -1.167 | 0.031553 |
| *EFS* | -1.166 | 0.004532 |
| *NFKBIA* | -1.166 | 0.005398 |
| *TENT5C* | -1.163 | 0.039488 |
| *PIPOX* | -1.162 | 0.003542 |
| *TSPAN5* | -1.162 | 0.006943 |
| *INAVA* | -1.160 | 0.005666 |
| *ABLIM3* | -1.160 | 0.033103 |
| *PPP1R3E* | -1.158 | 0.003770 |
| *CDCA8* | -1.158 | 0.014445 |
| *NAP1L5* | -1.158 | 0.045184 |
| *DCLK2* | -1.158 | 0.037648 |
| *DDRGK1* | -1.158 | 0.014803 |
| *MUC16* | -1.156 | 0.047306 |
| *AGAP9* | -1.156 | 0.040016 |
| *NFATC4* | -1.155 | 0.009510 |
| *TAS1R3* | -1.155 | 0.004181 |
| *CAB39L* | -1.153 | 0.030330 |
| *NET1* | -1.153 | 0.011302 |
| *CELF6* | -1.151 | 0.024046 |
| *EPHB6* | -1.150 | 0.010863 |
| *ESPL1* | -1.149 | 0.001475 |
| *OSBPL7* | -1.148 | 0.040400 |
| *CENPB* | -1.148 | 0.031031 |
| *PKIA* | -1.147 | 0.003623 |
| *TNP1* | -1.146 | 0.025200 |
| *TMTC1* | -1.146 | 0.021656 |
| *RASA4B* | -1.144 | 0.016447 |
| *HERC6* | -1.143 | 0.002236 |
| *BCL2L11* | -1.143 | 0.004175 |
| *CDC7* | -1.142 | 0.006329 |
| *PYROXD2* | -1.141 | 0.021991 |
| *KCNK1* | -1.140 | 0.003410 |
| *TIPARP* | -1.140 | 0.003242 |
| *MANEAL* | -1.140 | 0.007787 |
| *PRSS50* | -1.139 | 0.031454 |
| *MXD1* | -1.139 | 0.025131 |
| *ZNF677* | -1.138 | 0.004461 |
| *PRDM5* | -1.138 | 0.003082 |
| *TSPOAP1* | -1.138 | 0.012591 |
| *CCDC51* | -1.137 | 0.014794 |
| *ERAP2* | -1.137 | 0.022797 |
| *CD36* | -1.136 | 0.024288 |
| *PGBD5* | -1.135 | 0.001688 |
| *ZNF589* | -1.135 | 0.007098 |
| *CXXC4* | -1.135 | 0.005809 |
| *PPFIA3* | -1.135 | 0.003804 |
| *KCNH3* | -1.135 | 0.003162 |
| *TNFSF13* | -1.134 | 0.007644 |
| *STX1A* | -1.133 | 0.005714 |
| *CYP2J2* | -1.133 | 0.001703 |
| *ABHD17C* | -1.133 | 0.007580 |
| *DENND6B* | -1.133 | 0.008980 |
| *NEURL1* | -1.133 | 0.001661 |
| *TP53I11* | -1.132 | 0.002984 |
| *UBALD2* | -1.131 | 0.001977 |
| *TENT5A* | -1.131 | 0.039769 |
| *YPEL3* | -1.127 | 0.023515 |
| *H1-0* | -1.127 | 0.007382 |
| *FAM111B* | -1.126 | 0.004474 |
| *FYCO1* | -1.126 | 0.012252 |
| *STK35* | -1.125 | 0.004519 |
| *NAPB* | -1.125 | 0.005449 |
| *ZNF747* | -1.123 | 0.003309 |
| *TMEM238* | -1.123 | 0.030335 |
| *TSPAN2* | -1.122 | 0.019874 |
| *MTURN* | -1.122 | 0.009915 |
| *NOD1* | -1.121 | 0.002754 |
| *PARP12* | -1.121 | 0.004177 |
| *SFR1* | -1.121 | 0.003083 |
| *SAT1* | -1.119 | 0.004349 |
| *ZIK1* | -1.117 | 0.012928 |
| *PRRG3* | -1.116 | 0.003458 |
| *FRMPD3* | -1.113 | 0.002883 |
| *CFAP70* | -1.112 | 0.020633 |
| *AP5S1* | -1.111 | 0.002061 |
| *PCLO* | -1.111 | 0.032656 |
| *TMPRSS3* | -1.110 | 0.048472 |
| *BGLAP* | -1.110 | 0.011874 |
| *CA8* | -1.110 | 0.048710 |
| *MUC15* | -1.110 | 0.041553 |
| *NXT2* | -1.110 | 0.008700 |
| *FKBP1A* | -1.107 | 0.003741 |
| *ST18* | -1.107 | 0.014267 |
| *HLA-DQB1* | -1.107 | 0.019533 |
| *DYNLRB2* | -1.105 | 0.042977 |
| *BCO1* | -1.105 | 0.014861 |
| *SAPCD2* | -1.104 | 0.001975 |
| *CSNK2A3* | -1.104 | 0.017902 |
| *SPAG16* | -1.103 | 0.005831 |
| *SATB1* | -1.103 | 0.003608 |
| *CENPM* | -1.103 | 0.012549 |
| *FAM13A* | -1.102 | 0.001988 |
| *TMEM205* | -1.102 | 0.002802 |
| *GTF2IRD2* | -1.100 | 0.007866 |
| *FANCE* | -1.100 | 0.010834 |
| *PTGDR2* | -1.100 | 0.005320 |
| *IQCN* | -1.099 | 0.032183 |
| *MAOA* | -1.097 | 0.004888 |
| *C1orf74* | -1.096 | 0.004851 |
| *IFIH1* | -1.095 | 0.026572 |
| *CXCL16* | -1.093 | 0.025421 |
| *LGALS3* | -1.092 | 0.021948 |
| *TEKT2* | -1.092 | 0.024026 |
| *RORA* | -1.090 | 0.013971 |
| *PLCL1* | -1.090 | 0.003464 |
| *RAB15* | -1.089 | 0.002777 |
| *NBEA* | -1.088 | 0.012756 |
| *FFAR2* | -1.088 | 0.004623 |
| *SH2D3A* | -1.088 | 0.003981 |
| *DOK7* | -1.087 | 0.014936 |
| *CYB561* | -1.086 | 0.005159 |
| *NOTCH2NLA* | -1.085 | 0.001840 |
| *TNIP1* | -1.085 | 0.002604 |
| *GUCA2A* | -1.085 | 0.018723 |
| *TMC8* | -1.085 | 0.019826 |
| *DHX58* | -1.083 | 0.010420 |
| *FAM95C* | -1.083 | 0.029638 |
| *PLEKHA7* | -1.083 | 0.033197 |
| *NIPAL3* | -1.082 | 0.038081 |
| *GAPVD1* | -1.082 | 0.002471 |
| *TJP3* | -1.081 | 0.001974 |
| *HLA-DOA* | -1.081 | 0.022698 |
| *DMTN* | -1.081 | 0.003152 |
| *ANKRD27* | -1.081 | 0.011375 |
| *KCNK17* | -1.080 | 0.003792 |
| *IRF7* | -1.080 | 0.039550 |
| *HLA-DMA* | -1.079 | 0.023634 |
| *AKAP12* | -1.078 | 0.031537 |
| *C2CD4A* | -1.078 | 0.030742 |
| *TLCD2* | -1.077 | 0.017456 |
| *TUBA4A* | -1.074 | 0.019704 |
| *RASAL1* | -1.074 | 0.009397 |
| *SI* | -1.073 | 0.021445 |
| *PPP1R32* | -1.072 | 0.019605 |
| *ICOSLG* | -1.072 | 0.008046 |
| *GINS1* | -1.071 | 0.039429 |
| *ALCAM* | -1.071 | 0.017525 |
| *BCL6* | -1.071 | 0.026053 |
| *FOXA1* | -1.070 | 0.004061 |
| *TLR3* | -1.068 | 0.006402 |
| *TOM1L2* | -1.067 | 0.002647 |
| *HLA-B* | -1.066 | 0.022461 |
| *CARD6* | -1.066 | 0.045003 |
| *PIGA* | -1.066 | 0.005783 |
| *GZF1* | -1.064 | 0.002542 |
| *ELF4* | -1.063 | 0.002897 |
| *ANO1* | -1.062 | 0.010913 |
| *CAMKK1* | -1.062 | 0.014468 |
| *WDR90* | -1.062 | 0.037748 |
| *TP73* | -1.060 | 0.003336 |
| *CMTM8* | -1.059 | 0.027066 |
| *PSMF1* | -1.059 | 0.005417 |
| *BICDL1* | -1.059 | 0.046845 |
| *SOWAHB* | -1.059 | 0.012329 |
| *PPL* | -1.058 | 0.006826 |
| *RABGAP1L* | -1.058 | 0.005737 |
| *NYNRIN* | -1.057 | 0.010082 |
| *LYNX1* | -1.056 | 0.004470 |
| *IL17C* | -1.056 | 0.013912 |
| *ARRB2* | -1.055 | 0.002581 |
| *DLX3* | -1.053 | 0.007630 |
| *S100A16* | -1.053 | 0.022403 |
| *SEPTIN5* | -1.053 | 0.019551 |
| *IFI35* | -1.052 | 0.002066 |
| *H4C15* | -1.051 | 0.017775 |
| *PPM1L* | -1.051 | 0.044578 |
| *SPTB* | -1.051 | 0.002942 |
| *ZNF232* | -1.050 | 0.003169 |
| *OSBPL10* | -1.050 | 0.002895 |
| *PARP10* | -1.048 | 0.007827 |
| *CNKSR1* | -1.048 | 0.035606 |
| *COMTD1* | -1.048 | 0.017919 |
| *RIMBP2* | -1.048 | 0.004973 |
| *MAPK3* | -1.047 | 0.002777 |
| *OLFML2A* | -1.046 | 0.016444 |
| *DTX2* | -1.044 | 0.005688 |
| *AKR1C2* | -1.044 | 0.007760 |
| *MAPK15* | -1.042 | 0.003379 |
| *PCDHGA3* | -1.042 | 0.002507 |
| *CASP7* | -1.042 | 0.008853 |
| *TAC3* | -1.042 | 0.025088 |
| *USP53* | -1.041 | 0.048687 |
| *KIF24* | -1.040 | 0.002417 |
| *NAALADL2* | -1.039 | 0.035220 |
| *ZNF98* | -1.039 | 0.003560 |
| *CBX6* | -1.038 | 0.033778 |
| *PPFIBP2* | -1.038 | 0.020609 |
| *PDE4A* | -1.037 | 0.004173 |
| *EMP1* | -1.037 | 0.013589 |
| *NPY5R* | -1.037 | 0.020334 |
| *DDX11* | -1.036 | 0.020608 |
| *VSTM5* | -1.035 | 0.004869 |
| *ZNF816-ZNF321P* | -1.035 | 0.006324 |
| *TRERF1* | -1.034 | 0.005835 |
| *ITIH4* | -1.034 | 0.013320 |
| *ESAM* | -1.033 | 0.026807 |
| *UBOX5* | -1.033 | 0.015142 |
| *LTB4R* | -1.032 | 0.044307 |
| *ERMAP* | -1.032 | 0.024775 |
| *FREM2* | -1.032 | 0.027608 |
| *RALGAPA1* | -1.031 | 0.025632 |
| *BAIAP2L1* | -1.031 | 0.023945 |
| *ACBD7* | -1.031 | 0.033720 |
| *MUTYH* | -1.031 | 0.028227 |
| *SYCP2* | -1.030 | 0.015361 |
| *PROCA1* | -1.030 | 0.030550 |
| *USP43* | -1.030 | 0.002844 |
| *CNTNAP3B* | -1.028 | 0.020015 |
| *AKNA* | -1.028 | 0.009663 |
| *MCM8* | -1.028 | 0.005665 |
| *KRTCAP3* | -1.028 | 0.003431 |
| *BCL3* | -1.028 | 0.019358 |
| *NFIB* | -1.028 | 0.007351 |
| *SNPH* | -1.027 | 0.029018 |
| *CCDC57* | -1.027 | 0.029050 |
| *CNGA4* | -1.027 | 0.002497 |
| *NBPF10* | -1.026 | 0.039699 |
| *PAK6* | -1.025 | 0.003863 |
| *MT1F* | -1.023 | 0.007834 |
| *STXBP5L* | -1.023 | 0.027422 |
| *TBC1D30* | -1.020 | 0.029165 |
| *KRT15* | -1.018 | 0.021203 |
| *USP18* | -1.018 | 0.008775 |
| *ENO4* | -1.017 | 0.044005 |
| *IKZF1* | -1.016 | 0.036303 |
| *KLHL26* | -1.016 | 0.012073 |
| *DGKE* | -1.016 | 0.005883 |
| *PPP1R15A* | -1.015 | 0.003252 |
| *SRGAP1* | -1.015 | 0.027961 |
| *IZUMO4* | -1.015 | 0.004785 |
| *IAPP* | -1.015 | 0.018731 |
| *CDC25B* | -1.013 | 0.011099 |
| *ZNF337* | -1.013 | 0.048963 |
| *SPOPL* | -1.012 | 0.007724 |
| *CDKL5* | -1.011 | 0.043983 |
| *SCGB3A1* | -1.011 | 0.042434 |
| *KRT19* | -1.009 | 0.003439 |
| *NUP210* | -1.009 | 0.035957 |
| *SEC23B* | -1.008 | 0.007018 |
| *LLGL2* | -1.008 | 0.009128 |
| *PLP2* | -1.008 | 0.011470 |
| *CCDC159* | -1.007 | 0.027061 |
| *TICAM1* | -1.007 | 0.047406 |
| *FBXO32* | -1.006 | 0.002379 |
| *NBPF19* | -1.006 | 0.023508 |
| *PRXL2B* | -1.005 | 0.010893 |
| *DTD1* | -1.005 | 0.048617 |
| *ADRA2B* | -1.003 | 0.003941 |
| *CTSS* | -1.003 | 0.008804 |
| *SPATA12* | -1.002 | 0.018480 |
| *PLCB4* | -1.002 | 0.004344 |
| *PCDHGA7* | -1.002 | 0.003411 |
| *DUSP7* | -1.001 | 0.044091 |
| *RGS11* | -1.001 | 0.019069 |
| *ZNF880* | -1.001 | 0.003628 |
