# Supplementary material for: Identifying miRNA Signatures Associated with Pancreatic Islet Dysfunction in a FOXA2-Deficient iPSC Model
Source: Stem Cell Rev Rep. 2024 Jun 25;20(7):1915–31. doi: 10.1007/s12015-024-10752-0 (PMC11445299; doi:10.1007/s12015-024-10752-0)
Supplement: Supplementary file 5 — Supplementary Material 5 [file 12015_2024_10752_MOESM5_ESM.docx]

**Supplementary Table 5.** Top upregulated DEmiRs in *FOXA2^–/–^* islets compared with WT-islets (Log2 FC > 1, *P* < 0.05).

| **miRNA ID** | **Log2 FC** | ***P*-value** |
| --- | --- | --- |
| hsa-miR-199a-5p | 6.29761 | 0.000148 |
| hsa-miR-214-3p | 5.76148 | 0.000232 |
| hsa-miR-10a-5p | 4.66772 | 0.000003 |
| hsa-miR-214-5p | 4.58377 | 0.000730 |
| hsa-miR-199a-3p | 4.28411 | 0.001016 |
| hsa-miR-204-5p | 3.81157 | 0.012544 |
| hsa-miR-490-3p | 3.74824 | 0.000203 |
| hsa-miR-133a-5p | 3.14915 | 0.000027 |
| hsa-miR-122-5p | 3.09211 | 0.004089 |
| hsa-miR-133a-3p | 2.95520 | 0.000029 |
| hsa-miR-1-3p | 2.92150 | 0.000014 |
| hsa-miR-383-5p | 2.66077 | 0.002652 |
| hsa-miR-10b-5p | 2.64874 | 0.000668 |
| hsa-miR-618 | 2.57778 | 0.000094 |
| hsa-miR-196b-5p | 2.36430 | 0.000054 |
| hsa-miR-133b | 2.32345 | 0.000464 |
| hsa-miR-125b-1-3p | 2.29296 | 0.003248 |
| hsa-miR-218-5p | 2.16414 | 0.000457 |
| hsa-miR-145-5p | 2.10212 | 0.009372 |
| hsa-miR-145-3p | 2.06116 | 0.001509 |
| hsa-miR-365a-5p | 2.02153 | 0.000383 |
| hsa-miR-206 | 1.88778 | 0.005863 |
| hsa-miR-372-3p | 1.88276 | 0.000215 |
| hsa-miR-100-5p | 1.87694 | 0.021955 |
| hsa-miR-504-5p | 1.82716 | 0.000288 |
| hsa-miR-2682-5p | 1.82199 | 0.000314 |
| hsa-miR-373-3p | 1.76612 | 0.000992 |
| hsa-miR-143-3p | 1.69133 | 0.023539 |
| hsa-miR-371a-5p | 1.63440 | 0.002390 |
| hsa-miR-137-3p | 1.63327 | 0.005341 |
| hsa-miR-371a-3p | 1.62716 | 0.000863 |
| hsa-miR-152-5p | 1.61188 | 0.003248 |
| hsa-miR-10a-3p | 1.54962 | 0.001363 |
| hsa-miR-155-5p | 1.46370 | 0.001120 |
| hsa-miR-105-5p | 1.43233 | 0.021987 |
| hsa-miR-129-5p | 1.41903 | 0.007961 |
| hsa-miR-1269b | 1.41806 | 0.003371 |
| hsa-miR-1263 | 1.38661 | 0.006575 |
| hsa-miR-3085-3p | 1.38094 | 0.002291 |
| hsa-miR-708-5p | 1.38026 | 0.001880 |
| hsa-miR-106a-3p | 1.36400 | 0.035987 |
| hsa-miR-585-3p | 1.34695 | 0.002726 |
| hsa-miR-9983-3p | 1.30991 | 0.012296 |
| hsa-miR-193b-3p | 1.28944 | 0.017239 |
| hsa-miR-3117-3p | 1.28800 | 0.018137 |
| hsa-miR-6815-5p | 1.26580 | 0.007245 |
| hsa-miR-30b-3p | 1.23908 | 0.003385 |
| hsa-miR-1251-3p | 1.20758 | 0.021147 |
| hsa-miR-548w | 1.19917 | 0.004609 |
| hsa-miR-760 | 1.16573 | 0.012776 |
| hsa-miR-193b-5p | 1.14683 | 0.003133 |
| hsa-miR-10401-3p | 1.13964 | 0.031666 |
| hsa-miR-6843-3p | 1.13868 | 0.031624 |
| hsa-miR-548ah-3p | 1.13642 | 0.043918 |
| hsa-miR-365a-3p | 1.12864 | 0.006508 |
| hsa-miR-129-2-3p | 1.11738 | 0.017599 |
| hsa-miR-514a-3p | 1.09436 | 0.016005 |
| hsa-miR-489-3p | 1.07911 | 0.003577 |
| hsa-miR-887-5p | 1.05318 | 0.005964 |
| hsa-miR-34b-3p | 1.04424 | 0.022495 |
| hsa-miR-887-3p | 1.03743 | 0.027094 |
