# Supplementary material for: Identifying miRNA Signatures Associated with Pancreatic Islet Dysfunction in a FOXA2-Deficient iPSC Model
Source: Stem Cell Rev Rep. 2024 Jun 25;20(7):1915–31. doi: 10.1007/s12015-024-10752-0 (PMC11445299; doi:10.1007/s12015-024-10752-0)
Supplement: Supplementary file 6 — Supplementary Material 6 [file 12015_2024_10752_MOESM6_ESM.docx]

**Supplementary Table 6.** Top downregulated DEmiRs in *FOXA2^–/–^* islets compared with WT-islets (Log2 FC < −1, *P* < 0.05).

| **miRNA ID** | **Log2 FC** | ***P*-value** |
| --- | --- | --- |
| hsa-let-7d-5p | -4.18566 | 0.00017 |
| hsa-miR-98-5p | -3.79267 | 0.00057 |
| hsa-let-7d-3p | -3.69696 | 0.00025 |
| hsa-let-7i-5p | -3.55578 | 0.00099 |
| hsa-let-7g-5p | -3.48416 | 0.00021 |
| hsa-miR-146a-5p | -3.46109 | 0.00001 |
| hsa-let-7c-5p | -3.35395 | 0.00036 |
| hsa-let-7b-5p | -3.33790 | 0.00055 |
| hsa-miR-3059-5p | -3.29272 | 0.00015 |
| hsa-miR-551b-5p | -3.07552 | 0.00003 |
| hsa-let-7f-5p | -3.00945 | 0.00043 |
| hsa-miR-203b-3p | -2.87888 | 0.00002 |
| hsa-miR-551b-3p | -2.86735 | 0.00015 |
| hsa-miR-127-3p | -2.78930 | 0.00172 |
| hsa-let-7f-1-3p | -2.66048 | 0.00217 |
| hsa-let-7f-2-3p | -2.50539 | 0.00827 |
| hsa-miR-136-3p | -2.47541 | 0.00301 |
| hsa-miR-433-3p | -2.41518 | 0.00417 |
| hsa-let-7a-5p | -2.39032 | 0.00106 |
| hsa-miR-934 | -2.38368 | 0.00027 |
| hsa-let-7a-3p | -2.37988 | 0.00250 |
| hsa-let-7c-3p | -2.34103 | 0.00145 |
| hsa-miR-493-5p | -2.29658 | 0.00725 |
| hsa-miR-370-3p | -2.28731 | 0.00262 |
| hsa-miR-127-5p | -2.25616 | 0.00232 |
| hsa-miR-493-3p | -2.15845 | 0.00375 |
| hsa-miR-411-5p | -2.13715 | 0.00126 |
| hsa-miR-409-5p | -2.12795 | 0.00445 |
| hsa-miR-487b-3p | -2.11730 | 0.00365 |
| hsa-miR-494-3p | -2.11026 | 0.00703 |
| hsa-miR-382-3p | -2.11023 | 0.00981 |
| hsa-miR-412-5p | -2.09312 | 0.00037 |
| hsa-miR-382-5p | -2.08008 | 0.00453 |
| hsa-miR-381-3p | -2.06745 | 0.00549 |
| hsa-miR-432-5p | -2.04222 | 0.00370 |
| hsa-let-7b-3p | -2.02456 | 0.00311 |
| hsa-miR-873-3p | -2.01125 | 0.00049 |
| hsa-miR-409-3p | -2.00956 | 0.01033 |
| hsa-miR-134-5p | -1.98735 | 0.00342 |
| hsa-miR-654-5p | -1.93030 | 0.00616 |
| hsa-miR-99a-3p | -1.89894 | 0.00349 |
| hsa-miR-323a-3p | -1.87578 | 0.00520 |
| hsa-miR-873-5p | -1.85647 | 0.00036 |
| hsa-miR-495-3p | -1.84195 | 0.00700 |
| hsa-miR-655-3p | -1.81740 | 0.00128 |
| hsa-miR-487a-5p | -1.79663 | 0.00149 |
| hsa-miR-543 | -1.76665 | 0.00359 |
| hsa-miR-200a-3p | -1.76574 | 0.00061 |
| hsa-miR-1224-3p | -1.75385 | 0.00473 |
| hsa-miR-375-3p | -1.70244 | 0.01426 |
| hsa-miR-98-3p | -1.69519 | 0.00722 |
| hsa-miR-539-3p | -1.69198 | 0.00620 |
| hsa-miR-485-3p | -1.67730 | 0.00407 |
| hsa-miR-642a-5p | -1.58584 | 0.00374 |
| hsa-miR-625-3p | -1.56552 | 0.00053 |
| hsa-miR-431-3p | -1.56424 | 0.00120 |
| hsa-let-7i-3p | -1.56051 | 0.01407 |
| hsa-miR-429 | -1.54112 | 0.00101 |
| hsa-miR-141-3p | -1.52582 | 0.00084 |
| hsa-miR-203a-3p | -1.51655 | 0.03505 |
| hsa-miR-758-3p | -1.48987 | 0.01376 |
| hsa-let-7e-5p | -1.46834 | 0.00342 |
| hsa-miR-4510 | -1.46180 | 0.01151 |
| hsa-miR-876-3p | -1.43503 | 0.01689 |
| hsa-miR-668-3p | -1.42099 | 0.00443 |
| hsa-miR-182-3p | -1.38475 | 0.00920 |
| hsa-miR-200b-3p | -1.38147 | 0.00193 |
| hsa-miR-889-3p | -1.37138 | 0.01140 |
| hsa-miR-485-5p | -1.37054 | 0.04171 |
| hsa-miR-125b-2-3p | -1.34979 | 0.00590 |
| hsa-miR-329-3p | -1.34386 | 0.03001 |
| hsa-miR-200b-5p | -1.32878 | 0.00189 |
| hsa-miR-1185-1-3p | -1.32778 | 0.02417 |
| hsa-miR-3934-5p | -1.29827 | 0.00676 |
| hsa-miR-891a-5p | -1.26090 | 0.00617 |
| hsa-miR-642a-3p | -1.23877 | 0.00540 |
| hsa-miR-31-5p | -1.23331 | 0.01249 |
| hsa-miR-539-5p | -1.22424 | 0.01525 |
| hsa-miR-340-5p | -1.17002 | 0.01578 |
| hsa-miR-542-3p | -1.15544 | 0.00960 |
| hsa-miR-1250-5p | -1.15045 | 0.00437 |
| hsa-miR-146b-3p | -1.13802 | 0.00789 |
| hsa-miR-154-3p | -1.12851 | 0.02181 |
| hsa-miR-21-3p | -1.12340 | 0.00495 |
| hsa-miR-200a-5p | -1.11795 | 0.00462 |
| hsa-miR-31-3p | -1.11183 | 0.00729 |
| hsa-miR-337-3p | -1.10772 | 0.00680 |
| hsa-miR-296-5p | -1.10493 | 0.00326 |
| hsa-miR-99a-5p | -1.09727 | 0.01150 |
| hsa-miR-32-5p | -1.08862 | 0.03895 |
| hsa-miR-301a-3p | -1.07225 | 0.04241 |
| hsa-miR-450a-5p | -1.07125 | 0.00751 |
| hsa-miR-376a-3p | -1.06003 | 0.02819 |
| hsa-miR-4517 | -1.05251 | 0.01394 |
| hsa-miR-181c-5p | -1.04678 | 0.00587 |
| hsa-miR-3177-3p | -1.04512 | 0.02936 |
| hsa-miR-4728-5p | -1.01159 | 0.01216 |
| hsa-miR-146b-5p | -1.01154 | 0.01420 |
| hsa-miR-892b | -1.00092 | 0.00963 |
