# Supplementary material for: Identifying miRNA Signatures Associated with Pancreatic Islet Dysfunction in a FOXA2-Deficient iPSC Model
Source: Stem Cell Rev Rep. 2024 Jun 25;20(7):1915–31. doi: 10.1007/s12015-024-10752-0 (PMC11445299; doi:10.1007/s12015-024-10752-0)
Supplement: Supplementary file 7 — Supplementary Material 7 [file 12015_2024_10752_MOESM7_ESM.docx]

**Supplementary Table 7.** Top downregulated DEmiRs (Log2 FC < −1, *P* < 0.05) and their predicted upregulated target DEGs (Log2 FC > 2, *P* < 0.05) associated with nervous system development in *FOXA2^–/–^* islets compared with WT-islets.

| **Upregulated miRNA** | **Log2 FC** | ***P*-value** | **Predicted target gene** | **Log2 FC** | ***P*-value** |
| --- | --- | --- | --- | --- | --- |
| hsa-miR-429 | -1.541 | 0.001015 | *RELN* | 5.035 | 0.00003 |
| hsa-miR-323a-3p | -1.876 | 0.005197 | *GABRP* | 4.446 | 0.00094 |
| hsa-miR-337-3p | -1.108 | 0.006802 |  |  |  |
| hsa-miR-146a-5p | -3.461 | 0.000007 | *WNT2B* | 4.067 | 0.00036 |
| hsa-miR-934 | -2.384 | 0.000274 |  |  |  |
| hsa-miR-1224-3p | -1.754 | 0.004729 |  |  |  |
| hsa-miR-4510 | -1.462 | 0.01151 |  |  |  |
| hsa-miR-485-5p | -1.371 | 0.041705 |  |  |  |
| hsa-miR-301a-3p | -1.072 | 0.042411 |  |  |  |
| hsa-miR-181c-5p | -1.047 | 0.005868 |  |  |  |
| hsa-miR-4728-5p | -1.012 | 0.012164 |  |  |  |
| hsa-let-7d-5p | -4.186 | 0.000168 | *LIN28A* | 4.004 | 0.00005 |
| hsa-miR-4510 | -1.462 | 0.01151 |  |  |  |
| hsa-miR-329-3p | -1.344 | 0.030007 |  |  |  |
| hsa-miR-296-5p | -1.105 | 0.00326 |  |  |  |
| hsa-miR-181c-5p | -1.047 | 0.005868 |  |  |  |
| hsa-miR-4728-5p | -1.012 | 0.012164 | *ADGRA2* | 3.913 | 0.00009 |
| hsa-miR-495-3p | -1.842 | 0.007002 | *ALKAL2* | 3.619 | 0.00004 |
| hsa-miR-655-3p | -1.817 | 0.001278 |  |  |  |
| hsa-miR-200a-3p | -1.766 | 0.000607 |  |  |  |
| hsa-miR-542-3p | -1.155 | 0.009603 |  |  |  |
| hsa-miR-32-5p | -1.089 | 0.038947 |  |  |  |
| hsa-miR-450a-5p | -1.071 | 0.00751 |  |  |  |
| hsa-miR-655-3p | -1.817 | 0.001278 | *BMP4* | 3.558 | 0.00020 |
| hsa-miR-200a-3p | -1.766 | 0.000607 | *OLIG3* | 3.439 | 0.00091 |
| hsa-miR-539-3p | -1.692 | 0.006197 |  |  |  |
| hsa-miR-375-3p | -1.702 | 0.014259 | *CXCL12* | 3.428 | 0.00310 |
| hsa-miR-668-3p | -1.421 | 0.004435 |  |  |  |
| hsa-miR-329-3p | -1.344 | 0.030007 |  |  |  |
| hsa-miR-31-5p | -1.233 | 0.012494 |  |  |  |
| hsa-miR-154-3p | -1.129 | 0.021808 |  |  |  |
| hsa-miR-301a-3p | -1.072 | 0.042411 |  |  |  |
| hsa-miR-450a-5p | -1.071 | 0.00751 |  |  |  |
| hsa-miR-4728-5p | -1.012 | 0.012164 |  |  |  |
| hsa-miR-654-5p | -1.93 | 0.006161 | *WNT6* | 3.256 | 0.01048 |
| hsa-miR-876-3p | -1.435 | 0.016891 |  |  |  |
| hsa-miR-4728-5p | -1.012 | 0.012164 |  |  |  |
| hsa-miR-892b | -1.001 | 0.00963 |  |  |  |
| hsa-miR-296-5p | -1.105 | 0.003260 | *SEMA6B* | 3.142 | 0.00040 |
| hsa-miR-4728-5p | -1.012 | 0.012164 |  |  |  |
| hsa-let-7d-5p | -4.186 | 0.000168 | *GDF6* | 3.087 | 0.00059 |
| hsa-let-7c-3p | -2.341 | 0.001446 |  |  |  |
| hsa-miR-493-5p | -2.297 | 0.007254 |  |  |  |
| hsa-miR-654-5p | -1.93 | 0.006161 |  |  |  |
| hsa-miR-1224-3p | -1.754 | 0.004729 |  |  |  |
| hsa-miR-4728-5p | -1.012 | 0.012164 |  |  |  |
| hsa-miR-411-5p | -2.137 | 0.001262 | *PTN* | 3.071 | 0.00068 |
| hsa-miR-625-3p | -1.566 | 0.000532 |  |  |  |
| hsa-miR-655-3p | -1.817 | 0.001278 | *EDNRB* | 3.07 | 0.00014 |
| hsa-miR-539-3p | -1.692 | 0.006197 |  |  |  |
| hsa-miR-668-3p | -1.421 | 0.004435 |  |  |  |
| hsa-miR-31-5p | -1.233 | 0.012494 |  |  |  |
| hsa-miR-340-5p | -1.17 | 0.015782 |  |  |  |
| hsa-let-7c-3p | -2.341 | 0.001446 | *HAPLN1* | 3.03 | 0.00047 |
| hsa-miR-432-5p | -2.042 | 0.0037 |  |  |  |
| hsa-miR-654-5p | -1.93 | 0.006161 |  |  |  |
| hsa-miR-625-3p | -1.566 | 0.000532 |  |  |  |
| hsa-miR-301a-3p | -1.072 | 0.042411 |  |  |  |
| hsa-miR-433-3p | -2.415 | 0.004174 | *GAP43* | 3.022 | 0.02036 |
| hsa-miR-375-3p | -1.702 | 0.014259 |  |  |  |
| hsa-miR-21-3p | -1.123 | 0.004947 |  |  |  |
| hsa-miR-32-5p | -1.089 | 0.038947 |  |  |  |
| hsa-miR-301a-3p | -1.072 | 0.042411 |  |  |  |
| hsa-miR-4728-5p | -1.012 | 0.012164 |  |  |  |
| hsa-miR-200a-3p | -1.766 | 0.000607 | *GABRA2* | 2.927 | 0.00079 |
| hsa-miR-539-3p | -1.692 | 0.006197 |  |  |  |
| hsa-miR-203a-3p | -1.517 | 0.035053 |  |  |  |
| hsa-miR-182-3p | -1.385 | 0.009204 |  |  |  |
| hsa-miR-200b-5p | -1.329 | 0.00189 |  |  |  |
| hsa-miR-539-5p | -1.224 | 0.015253 |  |  |  |
| hsa-miR-4728-5p | -1.012 | 0.012164 | *TFAP2B* | 2.867 | 0.00236 |
| hsa-miR-432-5p | -2.042 | 0.003700 | *NTRK2* | 2.861 | 0.00761 |
| hsa-miR-642a-5p | -1.586 | 0.003740 | *CTNNA2* | 2.852 | 0.00030 |
| hsa-let-7c-3p | -2.341 | 0.001446 | *HOXB2* | 2.841 | 0.00862 |
| hsa-miR-340-5p | -1.17 | 0.015782 |  |  |  |
| hsa-miR-337-3p | -1.108 | 0.006802 |  |  |  |
| hsa-let-7c-3p | -2.341 | 0.001446 | *GATA2* | 2.807 | 0.00119 |
| hsa-miR-493-5p | -2.297 | 0.007254 |  |  |  |
| hsa-miR-99a-3p | -1.899 | 0.003487 |  |  |  |
| hsa-miR-32-5p | -1.089 | 0.038947 |  |  |  |
| hsa-miR-32-5p | -1.089 | 0.038947 | *HAND2* | 2.789 | 0.00825 |
| hsa-let-7d-5p | -4.186 | 0.000168 | *FZD4* | 2.773 | 0.00006 |
| hsa-miR-495-3p | -1.842 | 0.007002 |  |  |  |
| hsa-miR-655-3p | -1.817 | 0.001278 |  |  |  |
| hsa-miR-31-5p | -1.233 | 0.012494 |  |  |  |
| hsa-miR-296-5p | -1.105 | 0.00326 |  |  |  |
| hsa-miR-99a-3p | -1.899 | 0.003487 | *SFRP1* | 2.668 | 0.00015 |
| hsa-miR-892b | -1.001 | 0.009630 |  |  |  |
| hsa-miR-494-3p | -2.11 | 0.007032 | *PCP4* | 2.651 | 0.00029 |
| hsa-miR-3934-5p | -1.298 | 0.006761 |  |  |  |
| hsa-miR-31-3p | -1.112 | 0.007288 |  |  |  |
| hsa-miR-382-3p | -2.11 | 0.009809 | *DDR2* | 2.639 | 0.00062 |
| hsa-miR-146a-5p | -3.461 | 0.000007 | *ROR1* | 2.588 | 0.00029 |
| hsa-miR-127-5p | -2.256 | 0.002322 |  |  |  |
| hsa-miR-873-3p | -2.011 | 0.000493 |  |  |  |
| hsa-miR-892b | -1.001 | 0.00963 |  |  |  |
| hsa-miR-429 | -1.541 | 0.001015 | *CNTFR* | 2.555 | 0.01953 |
| hsa-miR-4510 | -1.462 | 0.01151 |  |  |  |
| hsa-miR-296-5p | -1.105 | 0.00326 |  |  |  |
| hsa-miR-4728-5p | -1.012 | 0.012164 |  |  |  |
| hsa-miR-134-5p | -1.987 | 0.003423 | *MMP24* | 2.409 | 0.00079 |
| hsa-miR-542-3p | -1.155 | 0.009603 |  |  |  |
| hsa-let-7f-1-3p | -2.66 | 0.002172 | *FGF2* | 2.402 | 0.00015 |
| hsa-miR-493-5p | -2.297 | 0.007254 | *PMP22* | 2.283 | 0.00240 |
| hsa-miR-429 | -1.541 | 0.001015 |  |  |  |
| hsa-miR-4510 | -1.462 | 0.01151 |  |  |  |
| hsa-miR-485-5p | -1.371 | 0.041705 |  |  |  |
| hsa-miR-4728-5p | -1.012 | 0.012164 |  |  |  |
| hsa-let-7f-1-3p | -2.66 | 0.002172 | *EDNRA* | 2.22 | 0.00114 |
| hsa-let-7f-2-3p | -2.505 | 0.008274 |  |  |  |
| hsa-miR-539-3p | -1.692 | 0.006197 |  |  |  |
| hsa-miR-429 | -1.541 | 0.001015 |  |  |  |
| hsa-miR-4510 | -1.462 | 0.01151 |  |  |  |
| hsa-miR-125b-2-3p | -1.35 | 0.005899 |  |  |  |
| hsa-miR-200b-5p | -1.329 | 0.00189 |  |  |  |
| hsa-miR-642a-3p | -1.239 | 0.005401 |  |  |  |
| hsa-miR-21-3p | -1.123 | 0.004947 |  |  |  |
| hsa-miR-337-3p | -1.108 | 0.006802 |  |  |  |
| hsa-miR-4728-5p | -1.012 | 0.012164 |  |  |  |
| hsa-let-7f-1-3p | -2.66 | 0.002172 | *NR2F2* | 2.199 | 0.00065 |
| hsa-let-7f-2-3p | -2.505 | 0.008274 |  |  |  |
| hsa-miR-4728-5p | -1.012 | 0.012164 |  |  |  |
| hsa-miR-934 | -2.384 | 0.000274 | *NTNG1* | 2.192 | 0.00060 |
| hsa-miR-99a-3p | -1.899 | 0.003487 |  |  |  |
| hsa-miR-200a-3p | -1.766 | 0.000607 |  |  |  |
| hsa-miR-431-3p | -1.564 | 0.001198 |  |  |  |
| hsa-let-7d-5p | -4.186 | 0.000168 | *WNT9B* | 2.131 | 0.00133 |
| hsa-miR-4510 | -1.462 | 0.011510 |  |  |  |
| hsa-miR-296-5p | -1.105 | 0.003260 |  |  |  |
| hsa-miR-3177-3p | -1.045 | 0.029363 |  |  |  |
| hsa-miR-892b | -1.001 | 0.009630 |  |  |  |
| hsa-miR-429 | -1.541 | 0.001015 | *SLIT2* | 2.123 | 0.00090 |
| hsa-miR-4510 | -1.462 | 0.011510 |  |  |  |
| hsa-miR-182-3p | -1.385 | 0.009204 |  |  |  |
| hsa-let-7c-3p | -2.341 | 0.001446 | *FGF19* | 2.086 | 0.02645 |
| hsa-miR-654-5p | -1.93 | 0.006161 |  |  |  |
| hsa-miR-4510 | -1.462 | 0.011510 |  |  |  |
| hsa-miR-200b-5p | -1.329 | 0.001890 | *BEX1* | 2.061 | 0.00381 |
| hsa-miR-542-3p | -1.155 | 0.009603 |  |  |  |
| hsa-miR-4510 | -1.462 | 0.011510 | *PCDHGB7* | 2.061 | 0.00256 |
| hsa-miR-4728-5p | -1.012 | 0.012164 |  |  |  |
| hsa-miR-892b | -1.001 | 0.009630 |  |  |  |
