# Supplementary material for: Identifying miRNA Signatures Associated with Pancreatic Islet Dysfunction in a FOXA2-Deficient iPSC Model
Source: Stem Cell Rev Rep. 2024 Jun 25;20(7):1915–31. doi: 10.1007/s12015-024-10752-0 (PMC11445299; doi:10.1007/s12015-024-10752-0)
Supplement: Supplementary file 8 — Supplementary Material 8 [file 12015_2024_10752_MOESM8_ESM.docx]

**Supplementary Table 8.** Top downregulated DEmiRs (Log2 FC < −1, *P* < 0.05) and their predicted upregulated target DEGs (Log2 FC > 1, *P* < 0.05) associated with lipid metabolism in *FOXA2^–/–^* islets compared with WT-islets.

| **Upregulated miRNA** | **Log2 FC** | ***P*-value** | **Predicted target gene** | **Log2 FC** | ***P*-value** |
| --- | --- | --- | --- | --- | --- |
| hsa-miR-429 | -1.541 | 0.001015 | *APOC3* | 3.897 | 0.006166 |
| hsa-miR-4728-5p | -1.012 | 0.012164 |  |  |  |
| hsa-miR-654-5p | -1.93 | 0.006161 | *PDGFRB* | 3.035 | 0.000385 |
| hsa-miR-892b | -1.001 | 0.009630 |  |  |  |
| hsa-miR-146a-5p | -3.461 | 0.000007 | *PDGFRA* | 2.958 | 0.000049 |
| hsa-miR-301a-3p | -1.072 | 0.042411 |  |  |  |
| hsa-miR-487a-5p | -1.797 | 0.001493 | *PLA2G2A* | 2.781 | 0.012291 |
| hsa-miR-4510 | -1.462 | 0.011510 |  |  |  |
| hsa-miR-4728-5p | -1.012 | 0.012164 |  |  |  |
| hsa-miR-4510 | -1.462 | 0.011510 | *APOC2* | 2.535 | 0.008866 |
| hsa-miR-4728-5p | -1.012 | 0.012164 |  |  |  |
| hsa-let-7f-1-3p | -2.66 | 0.002172 | *FGF2* | 2.402 | 0.000148 |
| hsa-miR-31-5p | -1.233 | 0.012494 | *SERPINA5* | 2.376 | 0.002514 |
| hsa-miR-376a-3p | -1.06 | 0.028186 |  |  |  |
| hsa-let-7d-5p | -4.186 | 0.00017 | *HMOX1* | 2.34 | 0.002408 |
| hsa-miR-494-3p | -2.11 | 0.00703 |  |  |  |
| hsa-miR-873-5p | -1.856 | 0.00036 |  |  |  |
| hsa-miR-642a-5p | -1.586 | 0.00374 |  |  |  |
| hsa-miR-485-5p | -1.371 | 0.04171 |  |  |  |
| hsa-miR-539-3p | -1.692 | 0.006197 | *MTTP* | 2.339 | 0.011627 |
| hsa-miR-296-5p | -1.105 | 0.003260 |  |  |  |
| hsa-miR-134-5p | -1.987 | 0.003423 | *AKR1D1* | 2.047 | 0.021353 |
| hsa-miR-873-5p | -1.856 | 0.000360 |  |  |  |
| hsa-miR-146a-5p | -3.461 | 0.000007 | *SOAT2* | 1.95 | 0.000241 |
| hsa-miR-301a-3p | -1.072 | 0.042411 | *FABP1* | 1.817 | 0.009621 |
| hsa-miR-4517 | -1.053 | 0.013944 |  |  |  |
| hsa-miR-432-5p | -2.042 | 0.0037 | *NR5A2* | 1.754 | 0.005532 |
| hsa-miR-134-5p | -1.987 | 0.00342 |  |  |  |
| hsa-miR-655-3p | -1.817 | 0.00128 |  |  |  |
| hsa-miR-429 | -1.541 | 0.00102 |  |  |  |
| hsa-miR-4510 | -1.462 | 0.01151 |  |  |  |
| hsa-miR-31-5p | -1.233 | 0.01249 |  |  |  |
| hsa-miR-450a-5p | -1.071 | 0.00751 |  |  |  |
| hsa-miR-4728-5p | -1.012 | 0.01216 |  |  |  |
| hsa-miR-376a-3p | -1.06 | 0.02819 |  |  |  |
| hsa-miR-370-3p | -2.287 | 0.002624 | *PLA2G12B* | 1.577 | 0.020816 |
| hsa-miR-494-3p | -2.11 | 0.007032 |  |  |  |
| hsa-miR-668-3p | -1.421 | 0.009630 |  |  |  |
| hsa-miR-892b | -1.001 | 0.009630 |  |  |  |
| hsa-miR-4510 | -1.462 | 0.011510 | *CIDEC* | 1.559 | 0.005503 |
| hsa-miR-551b-3p | -2.867 | 0.000153 | *CCDC3* | 1.511 | 0.016466 |
| hsa-miR-1224-3p | -1.754 | 0.004729 | *CYP27A1* | 1.483 | 0.002474 |
| hsa-miR-4510 | -1.462 | 0.011510 |  |  |  |
| hsa-miR-296-5p | -1.105 | 0.003260 | *SCARB1* | 1.367 | 0.007276 |
| hsa-miR-296-5p | -1.105 | 0.003260 | *FGFR4* | 1.255 | 0.048591 |
| hsa-miR-3177-3p | -1.045 | 0.029363 |  |  |  |
| hsa-miR-4728-5p | -1.012 | 0.012164 |  |  |  |
| hsa-miR-181c-5p | -1.047 | 0.005868 | *FLT1* | 1.223 | 0.041628 |
| hsa-miR-892b | -1.001 | 0.009630 |  |  |  |
| hsa-miR-127-5p | -2.256 | 0.00232 | *RGN* | 1.204 | 0.011920 |
| hsa-miR-382-5p | -2.08 | 0.00453 |  |  |  |
| hsa-miR-203a-3p | -1.517 | 0.03505 |  |  |  |
| hsa-miR-485-5p | -1.371 | 0.04171 |  |  |  |
| hsa-miR-1224-3p | -1.754 | 0.004729 | *NR0B2* | 1.199 | 0.004540 |
| hsa-miR-539-3p | -1.692 | 0.006197 |  |  |  |
| hsa-miR-485-5p | -1.371 | 0.041705 |  |  |  |
| hsa-miR-301a-3p | -1.072 | 0.042411 | *LRP2* | 1.186 | 0.028674 |
| hsa-miR-4517 | -1.053 | 0.013944 | *NR1H4* | 1.101 | 0.003411 |
| hsa-miR-495-3p | -1.842 | 0.007002 | *SULT2A1* | 1.095 | 0.048243 |
| hsa-miR-4510 | -1.462 | 0.011510 | *FGFR1* | 1.008 | 0.014626 |
| hsa-miR-296-5p | -1.105 | 0.003260 |  |  |  |
| hsa-miR-4728-5p | -1.012 | 0.012164 |  |  |  |
| hsa-miR-493-5p | -2.297 | 0.007254 | *KIT* | 1.003 | 0.044687 |
| hsa-miR-494-3p | -2.11 | 0.007032 |  |  |  |
| hsa-miR-539-3p | -1.692 | 0.006197 |  |  |  |
