# Supplementary material for: Identifying miRNA Signatures Associated with Pancreatic Islet Dysfunction in a FOXA2-Deficient iPSC Model
Source: Stem Cell Rev Rep. 2024 Jun 25;20(7):1915–31. doi: 10.1007/s12015-024-10752-0 (PMC11445299; doi:10.1007/s12015-024-10752-0)
Supplement: Supplementary file 9 — Supplementary Material 9 [file 12015_2024_10752_MOESM9_ESM.docx]

**Supplementary Table 1.** Media formulations for pancreatic beta cell differentiation.

| **Stage** | **Basal media** |
| --- | --- |
| Stage 1 and 2 | MCDB 131 + 10 mM Glucose  1.5 g/L NaHCO_3_  2 mM Glutamax  1% Pen/Strep  0.5% fatty acid free bovine serum albumin |
| Stage 3 and 4 | DMEM +  4.5g/l (25 mM) glucose  1% Pen/Strep  1% B27 supplement without vitamin A |
| Stage 5 | MCDB 131 + 20 mM Glucose  1.5 g/L NaHCO_3_  2 mM Glutamax  1% Pen/Strep  2% fatty acid free bovine serum albumin  1:200 Insulin-Transferrin-Selenium-X (ITS)  10 μg/ mL Heparin  10 μM Zinc Sulfate |
| Stage 6 | MCDB 131 + 20 mM Glucose  1.5 g/L NaHCO_3_  2 mM Glutamax  1% Pen/Strep  2% fatty acid free bovine serum albumin  1:200 Insulin-Transferrin-Selenium-X (ITS)  10 μg/ mL Heparin  10 μM Zinc Sulfate  1% non-Essential amino acids MEM-NEAA |
| Stage 7 | MCDB 131 +  1.5 g/L NaHCO_3_  2 mM Glutamax  1% Pen/Strep  2% fatty acid free bovine serum albumin  1:200 Insulin-Transferrin  10 μg/ mL Heparin  10 μM Zinc Sulfate  1% non-Essential amino acids MEM-NEAA  1 mM N-acetyl cysteine |

| **Stage** | **Cytokine** | **Final Concentration** |
| --- | --- | --- |
| Stage 1 day 1 | Activin A | 100 ng/ml |
|  | CHIR99021 | 3 μM |
|  | Rock inhibitor | 10 μM |
|  | Vitamin C | 0.25 mM |
| Stage 1 day 2-4 | Activin A | 100 ng/ml |
|  | Vitamin C | 0.25 mM |
| Stage 2 (2 days) | FGF10 | 50 ng/ml |
|  | LDN | 0.2 μM |
|  | WNT3a | 3 ng/ml |
|  | Vitamin C | 0.25 mM |
| Stage 3 (2 days) | LDN | 0.2 μM |
|  | FGF10 | 50 ng/ml |
|  | Retinoic acid | 2 μM |
|  | Sant-1 | 0.25 μM |
|  | Vitamin C | 0.25 mM |
| Stage 4 (4 days) | LDN | 0.2 μM |
|  | EGF | 100 ng/ml |
|  | Nicotinamide | 10 mM |
|  | Vitamin C | 0.25 mM |
| Stage 5 (3 days) | SANT1 | 0.25 μM |
|  | LDN | 0.1 μM |
|  | RA | 0.05 μM |
|  | Alk5i II | 10 μM |
|  | T3 | 1 μM |
|  | Vitamin C | 0.25 mM |
| Stage 6 (7 days) | LDN | 0.1 μM |
|  | Alk5i II | 10 μM |
|  | T3 | 1 μM |
|  | Gamma-secretase inhibitor | 1 μM |
|  | Vitamin C | 0.25 mM |
| Stage 7 (7-14 days) | Betacellulin | 20 ng/ mL |
|  | Alk5i II | 10 μM |
|  | T3 | 1 μM |
|  | Gamma-secretase inhibitor | 1 μM |
|  | Vitamin C | 0.25 mM |
